# Automated Phenotyping Unveils Trait‐Specific Genotypic Variation in Arbuscular Mycorrhiza Responsiveness in Maize (*Zea mays*)

**DOI:** 10.1111/pce.70709

**Published:** 2026-07-06

**Authors:** Florian Berger, Peter Muth, Wouter K. Vahl, Astrid Junker, Thomas Altmann, Stanislav Kopriva, Markus Herz, Caroline Gutjahr

**Affiliations:** ^1^ Plant Genetics TUM School of Life Sciences Technical University of Munich (TUM) Freising Germany; ^2^ Max‐Planck‐Institute of Molecular Plant Physiology Potsdam‐Golm Germany; ^3^ Faculty of Biology, Genetics, LMU Munich, Biocenter Martinsried Martinsried Germany; ^4^ Bavarian State Research Center for Agriculture, Institute for Crop Science and Plant Breeding Freising Germany; ^5^ Leibniz Institute of Plant Genetics and Crop Plant Research Seeland Germany; ^6^ Institute for Plant Sciences University of Cologne Cologne Germany

**Keywords:** arbuscular mycorrhiza, automated phenotyping, ionomics, maize, mycorrhiza responsiveness, plant nutrition, root symbiosis, root system growth, shoot growth, trait correlation

## Abstract

Arbuscular mycorrhiza (AM), a symbiosis between land plants and fungi that enhances plant mineral nutrition, has potential for application in sustainable agriculture. The extent to which plants benefit from the symbiosis depends on the plant‐fungal genotype combination, providing opportunities for breeding symbiosis‐optimized crops. Here, we analyzed several plant growth traits and shoot mineral element accumulation of 15 genetically diverse maize inbred lines in response to AM. Plants were grown in an automated phenotyping system, in which randomization produced reproducible shoot surface growth curves. We observed strong variation in AM responsiveness of shoot and root growth in a phosphate‐poor alkaline substrate. This substrate caused stunted growth in non‐inoculated control plants and a 165% to 633% increase in plant biomass in AM plants. We observed variation among the maize lines in the AM‐mediated increase in total shoot content of 24 mineral elements plus phosphate and sulphate. While nitrate accumulated in control plants, it was not detectable in the shoots of AM plants. Growth traits and accumulation of mineral elements were not uniformly correlated across the 15 maize lines. An exploratory ridge‐regression analysis showed that R_AM_ was only selectively predictable across traits, indicating that R_AM_ is mostly trait‐specific rather than uniformly coordinated across traits.

## Summary statement

A combination of automated, manual phenotyping and mass spectrometry examined growth traits and shoot nutrient accumulation of 15 maize lines in response to arbuscular mycorrhiza (AM). The detected variation in AM responsiveness (R_AM_) indicates that R_AM_ is only partly coordinated across traits.

## 1 Introduction

Plant productivity in agriculture is often limited by poor nutrient availability, which is normally compensated for by fertilizer application. However, pollution and greenhouse gas emissions resulting from extensive fertilization and fertilizer production demand solutions to reduce agricultural fertilizer use. The majority of land plant species, including many agriculturally important crops such as maize (*Zea mays*), form mutualistic symbioses with arbuscular mycorrhiza (AM) fungi of the phylum Glomeromycotina (Smith and Read 2008; Spatafora et al. 2016; Wang and Qiu 2006). These symbioses benefit plants with enhanced nutrient supply, especially in low nutrient substrates (Li et al. 2023; Miyata and Umehara 2024). AM fungi take up mineral nutrients from the soil via an extended extraradical hyphal network and transfer them into the root, where they are released from highly branched, tree‐shaped hyphal structures called arbuscules (Smith and Smith 2011; Wang et al. 2017). AM fungi also transfer water to the plant, although the mechanisms are less well understood (Kakouridis et al. 2022). In turn, they receive photosynthetically‐fixed carbon in the form of hexoses (Roth and Paszkowski 2017) and lipids (Keymer and Gutjahr 2018) from the plant host. Species belonging to the family of Glomeraceae also form vesicles, balloon‐like intraradical structures filled with lipids and nuclei that are hypothesized to serve for storage and as propagation units (Kameoka and Gutjahr 2022). Besides improving plant nutrient acquisition, AM symbiosis can also promote resistance against biotic and abiotic stresses (Wahab et al. 2023; Wang et al. 2024).

Through these benefits, AM can significantly improve plant health, growth and yield, and it has been shown that AM improves maize yield by 30% on phosphate‐poor soils (Ramirez‐Flores et al. 2020). Thus, optimized application of AM symbiosis in agriculture may stabilize yields despite reduced fertilizer application. Phosphorus (P) is thought to be the most relevant nutrient transferred by AM fungi as P is poorly accessible in most natural soils and therefore limiting for plant growth (Lynch 2011; Nouri et al. 2014). AM fungi are thought to increase phosphate uptake because their extensive hyphal networks explore soil volumes beyond the phosphate depletion zone, which forms around plant roots due to P uptake by the plant (Drew et al. 2003; Li et al. 1991). Furthermore, they collaborate with phosphate‐solubilizing hyphosphere bacteria, which release soluble phosphate from organic substrates (Wang et al. 2023).

AM formation is regulated by the plant according to its nutrient status. For example, at a high phosphate status, colonization is reduced because the master regulator of phosphate starvation PHOSPHATE STARVATION RESPONSE (PHR) is scavenged by phosphate‐sensing SPX proteins (Paries and Gutjahr 2023). However, the lack of other nutrients can partially rescue colonization, indicating that the transfer of other nutrients via AM fungi also plays a significant role in plant nutrition (Breuillin‐Sessoms et al. 2015; Nouri et al. 2014). For example, AM fungi are also important for nitrogen (N) acquisition (Breuillin‐Sessoms et al. 2015; Tian et al. 2023; Wang et al. 2020) and in the uptake of sulphur, potassium, zinc and iron (Allen and Shachar‐Hill 2009; Liu et al. 2019; Tamayo et al. 2018; Watts‐Williams et al. 2023). In turn, it has been reported that AM influences plant resilience against heavy metal stress. For example, AM fungi enhance sequestration of arsenic (As) and lead (Pb) in maize roots, thereby limiting their translocation to shoots (Bai et al. 2008; Sudová and Vosátka 2007; Zhan et al. 2018).

In nutrient‐depleted soils, many plants display stronger growth in the presence of AM than in its absence. The extent to which a plant responds to AM colonization is termed AM responsiveness (R_AM_) (Janos 2007; Sawers et al. 2010). R_AM_ can vary significantly among different plant species and among genotypes within the same species, providing opportunities for optimizing R_AM_ through breeding (Sawers et al. 2008). In addition, it is influenced by environmental factors and the plant and AM fungal species involved, making it a highly complex trait (Berger and Gutjahr 2021). In maize, the large genetic diversity within the species results in genotype‐specific R_AM_ that can range from strongly positive to neutral or even negative, depending on the genotype and environmental conditions (Kaeppler et al. 2000; Ramirez‐Flores et al. 2019; Ruiz‐Lozano et al. 2022; Sawers et al. 2017). For example, Kaeppler et al. (2000) demonstrated positive R_AM_ with strong variation among 28 lines at low P conditions, while for the same lines grown in a high P soil R_AM_ varied between weakly positive to weakly negative. Sawers et al. (2017) similarly identified positive, but strongly variable R_AM_ for 30 maize lines under P‐limiting conditions. In addition, they found that AM influenced the uptake of 19 nutrients and metal ions in a maize‐line‐specific manner and that changes in mineral uptake were coordinated for several sub‐sets of nutrients across lines and AM‐conditions (Ramirez‐Flores et al. 2017). For two maize lines, the AM‐mediated increase in uptake of 15 nutrients correlated with an AM‐stimulated increase in root system size. However, for 6 nutrients (B, Ca, Mg, P, S, Sr) AM had a significant additional effect on uptake that could not be explained by root size alone (Ramirez‐Flores et al. 2019). Together, this underscores that R_AM_ is complex, can be defined for different traits and can be a direct but also an indirect effect of AM (Ramirez‐Flores et al. 2019). This highlights the need for genotype comparisons to uncover genetic avenues for optimizing AM benefits in agricultural systems (Sawers et al. 2008).

The use of automated phenotyping platforms opens new opportunities for describing and understanding R_AM_, as it enables reproducible high‐resolution temporal imaging of plant growth under diverse environmental conditions (Nagel et al. 2012; Paez‐Garcia et al. 2015; Yang et al. 2020). These systems enable high‐throughput quantification of certain growth parameters as well as key physiological traits such as biomass accumulation, water use efficiency (WUE), root and/or leaf architecture, and can thereby provide detailed insights into the multifaceted interactions between AM fungi, plant genotypes, and environmental factors (Ngo et al. 2023; Watts‐Williams et al. 2019; Giovannini et al. 2025).

Despite recent advances, key questions remain about the genetic and physiological factors driving variation in R_AM_ among maize genotypes. In this study, we evaluated the response of 15 diverse maize lines to inoculation with the AM fungus *Rhizophagus irregularis*, assessing growth performance and nutrient uptake under both AM and control conditions. Nine of the lines (UH250, F353, UH304, F252, W117, D09, Mo17, F618 and D06) represent the diversity of the European Dent breeding pool and were used as parental lines from the EU‐nested association mapping (NAM) population (Bauer et al. 2013), thereby allowing for follow‐up QTL mapping. We also included the Flint inbred line UH007 which was used in the EU‐NAM to connect the Dent with the Flint pool (Bauer et al. 2013) along with two additional Dent inbred lines (Oh7b, Oh43), which showed one of the weakest and the strongest shoot growth R_AM_, respectively, in a previous study (Sawers et al. 2017) and the broadly used reference inbred lines B73, PH207 and W22 (Hirsch et al. 2016; McMullen et al. 2009). Using an automated phenotyping system, we captured detailed temporal growth data, in addition to manually determined plant biomass and the shoot ionome. We find beneficial effects of AM symbiosis across a range of maize genotypes for multiple traits. Additionally, we highlight the utility of image‐derived shoot area as a reliable proxy for biomass, offering a high‐throughput, labour‐efficient approach for non‐destructive phenotypic screening. By linking AM responsiveness with growth traits and nutrient acquisition, our findings provide important insights into the potential for optimizing AM associations in maize.

## 2 Materials and Methods

### 2.1 Plant and Fungal Material

We employed a panel of 15 maize (*Z. mays*) inbred lines, consisting of parental lines from the EU‐NAM population – including the Flint inbred line UH007 and the Dent inbred lines UH250, F353, UH304, F252, W117, D09, Mo17, F618 and D06 (Bauer et al. 2013) – along with two additional Dent inbred lines (Oh7b, Oh43), and the broadly used reference inbred lines B73, PH207 and W22 (Hirsch et al. 2016; McMullen et al. 2009). The lines were grown either in the presence or absence of the AM fungus *R. irregularis* DAOM197198 (‘Quality D’, Agronutrition, Toulouse, France).

### 2.2 Experimental Design

Experiment 1: A total of 180 plants (15 genotypes × 2 treatments × 6 replicates) was cultivated in 12 rows (15 pots each) using the LemnaTec ‘Moving Fields’ high‐throughput phenotyping system at the Institute for Plant Breeding and Crop Science (IPZ) of the Bavarian State Research Centre for Agriculture (LfL) in Freising, Germany. The system consisted of carrier platforms (Bosch Rexroth TS2plus 400 mm with WT2 carriers) on a conveyor belt, an automated weighing and watering station, and a ‘Scanalyzer 3D’ cabin for imaging. The phenotyping cabin (4.2 × 1.3 × 3.1 m) housed two RGB cameras (Basler piA2400‐17gm; 2456 × 2058 pixels), illuminated by fluorescent lamps (Osram HE 28 W/865). One camera and 14 lamps were mounted above the conveyor band; the second camera and 28 lamps were mounted horizontally. Both cameras were installed at 2.8 m distance to the conveyor belt. To minimize positional effects, starting positions were randomized and positions of pots were rotated daily.

Experiment 2: 32 F353 and 32 D06 plants (2 genotypes × 2 treatments × 16 replicates) were grown in a greenhouse using a LemnaTec system across one row with 32 carriers at the Leibniz Institute of Plant Genetics and Crop Plant Research (IPK) in Gatersleben (Junker et al. 2014). Each carrier held two replicates of the same genotype × treatment combination, with four carriers forming a block representing all genotype × treatment combinations.

Experiment 3: In this experiment, 36 B73 plants (6 harvesting time points × 1 genotype × 2 treatments × 3 replicates) were grown in the Greenhouse Facility (Plant Technology Centre, Dürnast) of the Technical University of Munich in Freising, Germany. Plants were completely randomized and grown in a single row.

### 2.3 Growth Conditions

The growth conditions for each experiment are described in Supporting Information S2: Materials and Methods 1.

### 2.4 Analysis of Shoot Traits Derived From Image Analysis

Images taken in experiment 1 were analyzed using the LemnaGrid software (LemnaTec GmbH, Aachen, Germany). For each image analyzed, the absolute number of pixels classified as a plant part was multiplied by the image scale (~0.73 mm/pixel) to yield the shoot surface area. This area was averaged over the four side‐view images per recording. In experiment 2, to extract image‐based shoot traits, the Integrated Analysis Platform software (version 2.0; Klukas et al. 2014) was used to process multiple side‐view RGB images with a customized analysis pipeline. For single‐plant analysis, 45° and 225° RGB images were tiled, and plant pixels were extracted for each of the two tiles separately.

### 2.5 Manual Growth Trait Measurements

In addition to recordings by the LemnaTech system, in experiment 1 growth traits were recorded manually and included plant height (soil to extended leaf tips), plant development stage (Biologische Bundesanstalt, Bundessortenamt und Chemische Industrie scale, BBCH) and chlorophyll content estimates (Chlorophyll Meter SPAD‐502 Plus, Konica Minolta Business Solutions, Langenhagen, Germany; average of four spots along the last fully developed leaf), as well as regular observations of heading date. Daily water supplies to pots were recorded, and the gross WUE was calculated, defined as the combined biomass dry weight of roots and shoots produced per litre of water. At 69 and 66 days after sowing (das), plants were harvested in experiments 1 and 2, respectively.

In experiment 3, green leaf area (gla) was measured weekly starting at 21 das. Gla is determined as a manually measured proxy of shoot area based on the multiplication of measurements of a given plant's green leaf length and width, multiplied by a factor of 0.73 based on (McCree and Davis 1974). Starting at 28 das, three AM and three control plants were harvested every week for fresh and dry weight measurements as well as quantification of root colonization by AM fungi (AMF) until the last plants were harvested at 63 das.

For fresh and dry weight measurements, plant shoots were removed 5 cm (experiment 1) or 2 cm (experiments 2 and 3) above the substrate surface, and roots were carefully washed in water and blotted dry with a towel. Fresh weight of both plant organs was recorded, and root samples for analysis of root colonization levels by the AMF were taken and stored in 10% potassium hydroxide (KOH). In experiment 1, plant tissue was pre‐dried at 35°C airflow for 7 to 15 days, followed by 48 h of oven drying at 70°C, before the dry weight of shoots and roots was recorded. In experiments 2 and 3, fresh plant tissue was dried in a drying cabinet at 60°C for 7 days before dry weights were recorded.

### 2.6 Estimation of Root Colonization by *R. irregularis*

The root system of each plant was sampled randomly from at least 10 separate regions. Root pieces were stored in 10% KOH until staining. Roots were stained with acid ink, and percent root length colonization by AMF structures was analyzed using the modified intersect method as described in Torabi et al. (2021). Ten sections per root piece and 10 root pieces per plant were quantified under a light microscope at 200× magnification and scored for the presence of AMF structures.

### 2.7 Analysis of the Shoot Ionome

After oven drying, whole plant shoots of experiment 1 were milled using a rotary mill with a 0.5 mm sieve (Brabender GmbH & Co KG, Duisburg, Germany). For anion measurements, dry plant material was homogenized in 1 mL of deionized water, and the anions (nitrate, phosphate and sulphate) were separated by the Dionex ICS‐1100 chromatography system on a Dionex IonPac AS22 RFIC 4 × 250 mm analytic column (Thermo Scientific, Darmstadt, Germany) with 4.5 mM NaCO_3_/1.4 mM NaHCO_3_ as running buffer (Dietzen et al. 2020). Concentration of mineral elements was determined by inductively coupled plasma mass spectrometry (ICP‐MS) from ca. 10 mg lyophilized tissues by the Biocenter MS Platform Cologne, University of Cologne, using an Agilent 7700 ICP‐MS (Agilent Technologies, Santa Clara, CA, USA; Almario et al. 2017).

For the analysis of N in shoot material, samples of dried shoots were re‐dried at 60°C for 24 h, ball‐milled to a fine powder, and 0.70 ± 0.05 mg aliquots were weighed into tin cups (3.3 × 5 mm; LüdiSwiss, Flawil, Switzerland). Samples were combusted in an elemental analyzer (NA 1110; Carlo Erba, Milan, Italy) interfaced via a Conflo III to a Delta Plus isotope ratio mass spectrometer (Finnigan MAT, Bremen, Germany) following Baca Cabrera et al. (2021). The absolute amount of nitrogen per sample (µg N) was calculated from the integrated *m/z* 28 (N_2_) peak area using the instrument‐specific response factor derived from the calibration curve. To ensure analytical accuracy and precision, a laboratory internal standard of known N content (wheat flour) was analyzed after every ten maize samples, and the measured %N for the standard was required to fall within its certified uncertainty range.

### 2.8 Statistical Analyses and Data Visualization

All statistical analyses, data handling and visualization were performed in R version 4.4.1 (R Core Team 2024). Details are described in Supporting Information S2: Materials and Methods 2.

### 2.9 Missing Values

Post‐experimental analysis revealed that two control plants (W117; UH007) were fully colonized with the AMF in experiment 1. These were analyzed as AM‐inoculated plants in all analyses. Four AM plants of the genotypes UH007 (2), UH250 (1) and B73 (1) showed weak growth, but were found to be fully colonized (71% to 100% total colonization) and were treated as AMF inoculated in the subsequent analyses. Ten plants were damaged mechanically in the course of the experiment and were excluded from all analyses (F618 AM, UH007 AM, UH007 control, D09 AM, Oh43 control, 2x F353 AM, F353 control, Oh7b control, W117 control). Due to problems during the analysis, two shoot samples could not be analyzed by ICP‐MS (Mo17 AM, D09 control).

## 3 Results

### 3.1 Automated Phenotyping Reveals Variation in Growth Responses to AM in a Panel of 14 Dent and 1 Flint Maize Inbred Lines

To analyze AM responsiveness (R_AM_) across 15 maize inbred lines, we used an automated LemnaTec phenotyping system at the Landesanstalt für Landwirtschaft (LfL) in Freising, Germany (experiment 1; Supporting Information S2: Figure S1; Supporting Information S1: Movie S1). This enabled the non‐destructive acquisition of growth curves for 65 days by calculating the shoot area from images captured almost daily throughout the experiment. To better reveal differences in shoot area R_AM_ among lines, we grew the lines at very low nutrient availability and in the presence of a slightly alkaline (pH 7.6) sand‐clay (9:1) mixture. Thus, the growth of control plants stagnated significantly, while AM plants grew better with all 15 lines showing positive R_AM_. We observed considerable growth variation among AM plants across the inbred lines, while variation was much smaller in control plants, leading to a strong variation in R_AM_ (Figure 1A,B). To understand whether the image‐derived shoot area reflects shoot biomass, we determined the shoot dry weight of all plants at the end of the experiment (Figure 1C). The digitally determined shoot area strongly correlated with shoot dry weight (Figure 1D). Thus, the automated imaging system is suitable to make inferences about shoot biomass without the need for labour‐intensive manual measurements, by providing reasonable approximations.

**Figure 1:**
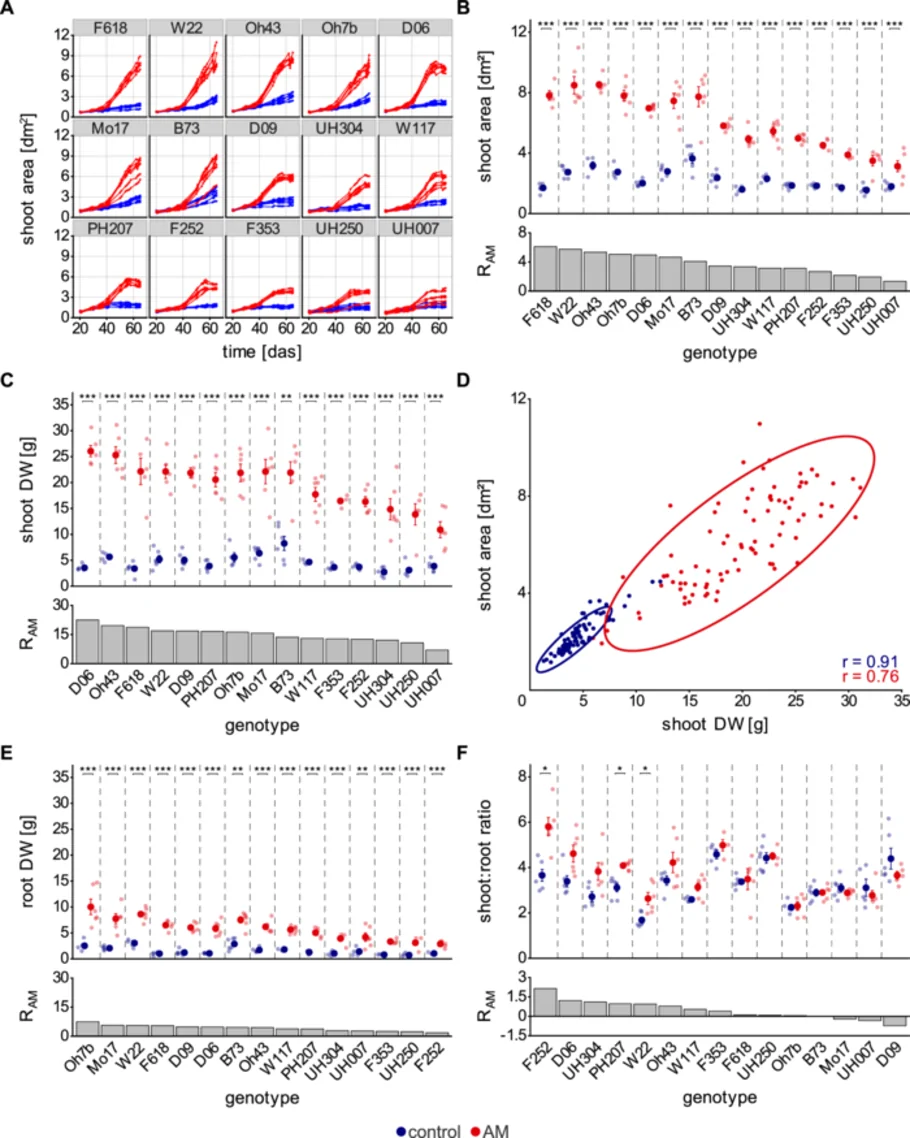
Fifteen maize genotypes vary in their growth response to arbuscular mycorrhiza. (A) Time‐course of image‐derived shoot areas recorded almost daily between 20 and 65 days after sowing (das) in a LemnaTec system. Plants were grown either with (AM; red lines) or without (control; blue lines) the arbuscular mycorrhiza fungus *Rhizophagus irregularis*. Each panel illustrates the shoot growth dynamics of one of the maize genotypes, ordered by the AM responsiveness (R_AM_; difference between the mean values of AM and control plants) at the end of the experiment. (B) Shoot area derived from imaging data, (C) shoot dry weight (DW), (E) root DW and (F) shoot:root ratio of maize genotypes measured at 69 das. The upper panel shows values from individual plants (light‐coloured dots) along with the means (dark‐coloured dots) and standard error of the mean for each genotype and treatment combination. Asterisks denote significant differences between treatment means within a genotype (*n* = 4–7, *t*‐test, *p < 0.05, **p < 0.01, ***p < 0.001). The lower panel shows genotype‐specific R_AM_. In all panels, genotypes are displayed in decreasing order of R_AM_. (D) Correlation between shoot area and shoot DW at 69 das for both AM (red) and control plants (blue). Pearson correlation coefficients are presented in each treatment's respective colour in the lower right corner. All data are from experiment 1.

The regular imaging throughout the experiment also allowed us to compare among genotypes the time point post‐inoculation at which the AM‐effect became statistically significant (based on model‐derived contrasts; 95% confidence level not overlapping zero). We observed substantial variation among genotypes, with differences between AM and control plants becoming apparent and statistically significant as early as 40 das (e.g., lines W22, Oh43, D06, Mo17, D09, F353) and as late as 50 das (e.g., lines B73, UH304, UH007, Supporting Information S2: Table S1). Although there were differences in the timing of R_AM_ becoming detectable, this did not necessarily determine the final R_AM_. Several genotypes showing early significant differences between AM and control plants were among the strongest responders, yet other lines exhibited weak responses despite early detectable R_AM_ (e.g., F353; Figure 1A,B and Supporting Information S2: Figure S2 and Table S1).

We selected two lines, D06 and F353, as examples with contrasting R_AM_ to examine whether their difference in R_AM_ could be replicated in another location (experiment 2, Supporting Information S2: Figure S3). Using a similar automated phenotyping system at the IPK Gatersleben (however, without plant randomization, explaining the stronger variability among replicate plants), we could reproduce the results obtained in experiment 1, and D06 had a stronger R_AM_ than F353 in both experiments (Figure 1A,B, Supporting Information S2: Figure S3A). The timepoints at which AM effects became significant were also very consistent between experiments, that is, for D06 at 40 das in Freising and 40 das in Gatersleben and for F353 at 40 das in Freising and at 42 das in Gatersleben (Supporting Information S2: Figure S4 and Table S2).

We conclude that endpoint R_AM_ is robust and reproducible under the controlled conditions of automated phenotyping platforms. The comparison between D06 and F353 is particularly insightful, as the lines differ markedly in their endpoint R_AM_, although they start showing significant R_AM_ at basically the same time point in two different experiments. This highlights that the time at which AM effects become significant is not necessarily a predictor of the amplitude of R_AM_.

### 3.2 R_AM_ of Shoot Growth Does Not Correlate With Endpoint Root Colonization Levels

To understand whether differences in R_AM_ of shoot growth may be caused by differences in root colonization among the lines, we quantified root length colonization across all genotypes (Supporting Information S2: Figure S5A,C,E). We found no significant differences in total root length colonization or colonization with arbuscules (Supporting Information S2: Figure S5A,C). We only found a reduced root colonization with vesicles for the two lines W22 and F252, which was significant in comparison to Oh7b (Supporting Information S2: Figure S5E). No correlation was observed between colonization parameters and shoot dry weight (Supporting Information S2: Figure S5B,D,F), indicating that the end‐point colonization does not explain differences in R_AM_ for this trait. However, the rate of colonization, which could not be assessed in this experiment, may have influenced growth, as faster colonization may have promoted growth at an earlier point in time.

Thus, we examined the relationship between colonization and growth over time for the line B73 as an example (experiment 3, Supporting Information S2: Material and Methods 1). We harvested plants inoculated with *R. irregularis* at 28 das and then every week for a 6‐week period (Supporting Information S2: Figure S6). Consistent with experiment 1, positive R_AM_ was observed for green leaf area, shoot dry weight, and root dry weight (Supporting Information S2: Figure S6A,B,D). R_AM_ was negative or neutral for all three traits at 28 das, but increased gradually over time. The difference between B73 AM and control plants was significant at 42 das. Colonization levels increased after 35 das, with a slight delay compared to green leaf area and shoot and root dry weight, reaching a plateau at 56 das (Supporting Information S2: Figure S6C). Further, total colonization was positively correlated with green leaf area (*r* = 0.73), shoot dry weight (*r* = 0. 67), and root dry weight (*r* = 0. 60; Supporting Information S2: Figure S7) and was also positively correlated with R_AM_ (Supporting Information S2: Table S3). Although AM plants were colonized by 28 das, colonization levels were still low (approx. 20%). A notable difference in plant growth between AM and control plants became evident as colonization levels increased to an average of 36% at 42 das. The positive R_AM_ continued to increase over the course of the experiment.

Thus, AM‐responsive growth and R_AM_ increase in parallel with rising colonization levels (at least for green leaf area, shoot and root dry weight). However, it remains unclear whether plant growth facilitates higher colonization levels or whether increased colonization drives enhanced plant growth. Further research is needed to determine the direction of this relationship.

### 3.3 AM Responsiveness Is Not Correlated Across All Traits

In addition to shoot dry weight, we also determined the root dry weight and found that the ranking of lines for root dry weight R_AM_ was similar to that for shoot dry weight (Figure 1C,E). Nevertheless, in two‐thirds of the genotypes AM caused a higher shoot:root ratio as compared to control plants (Figure 1F), indicating an enhanced allocation of resources to the shoot in these lines, likely due to improved nutrient acquisition facilitated by the fungus.

We also manually determined plant height, heading date, and growth stages according to the Biologische Bundesanstalt, Bundessortenamt und Chemische Industrie (BBCH) and Soil and Plant Analyser Development (SPAD)‐based chlorophyll content (Xiong et al. 2015). All lines responded to AM with increased plant height and showed similar variation in R_AM_ for shoot and root dry weight (Supporting Information S2: Figure S8A). According to BBCH scores, AM accelerated development starting from 44 das when data from all lines were combined (Supporting Information S2: Figure S9A), although when lines were analyzed separately, this was clearly visible for only about five genotypes and statistically significant for only two lines (D06 and F252; Supporting Information S2: Figures S8B,C and S9B). Correspondingly, all genotypes, except W22, showed a later heading date of control plants compared to AM plants while surprisingly the line Oh7b did not flower at all in the presence of AM (Supporting Information S2: Figure S8D). We hypothesize that the faster development of AM plants is driven by increased nutrient availability, such as P, while control plants, with stronger P deficiency, may experience delayed development and flowering (Ren et al. 2019). However, the ability to respond to AM with accelerated development is line‐specific, indicating variability in the genetic basis controlling this trait.

Chlorophyll content, measured as a proxy for photosynthetic activity, exhibited positive R_AM_ when all genotypes were analyzed together (Supporting Information S2: Figure S9C). When considered separately, all lines showed a significant increase in chlorophyll content at 44 das, with all but one genotype showing a significantly AM‐induced increase at 58 das, and only five lines showing this at 63 das (Supporting Information S2: Figures S8E,F and S9D), indicating that AM increases chlorophyll content, which may be levelled again during plant aging. Notably, the five genotypes with the highest R_AM_ for chlorophyll content were among the weakest responders in terms of shoot and root dry weight, aligning with a majorly negative correlation between chlorophyll content and root or shoot dry weight (Supporting Information S2: Table S4). This indicates that chlorophyll content R_AM_ is not uniformly coupled to growth R_AM_ across genotypes. In line with this, the exploratory predictive analysis showed that chlorophyll content R_AM_ was moderately predictable from other R_AM_ growth traits, but only weakly predictable from ionomic R_AM_ traits (see below), indicating a trait‐specific rather than uniform pattern of R_AM_ (Supporting Information S2: Table S5). Potentially, this could be due to a lack of mineral nutrients hindering some genotypes from converting potentially higher photosynthesis rates through higher chlorophyll content in AM plants into enhanced growth.

Overall, we observed substantial variation in R_AM_ across the tested genotypes for all measured traits. Furthermore, R_AM_ was strongly trait‐specific and not uniform across traits, leading to trait‐dependent changes in the ranking of genotypes based on R_AM_ and variation in R_AM_ reaching up to 400% from the mean in both directions for individual lines and traits (Figure 2).

**Figure 2:**
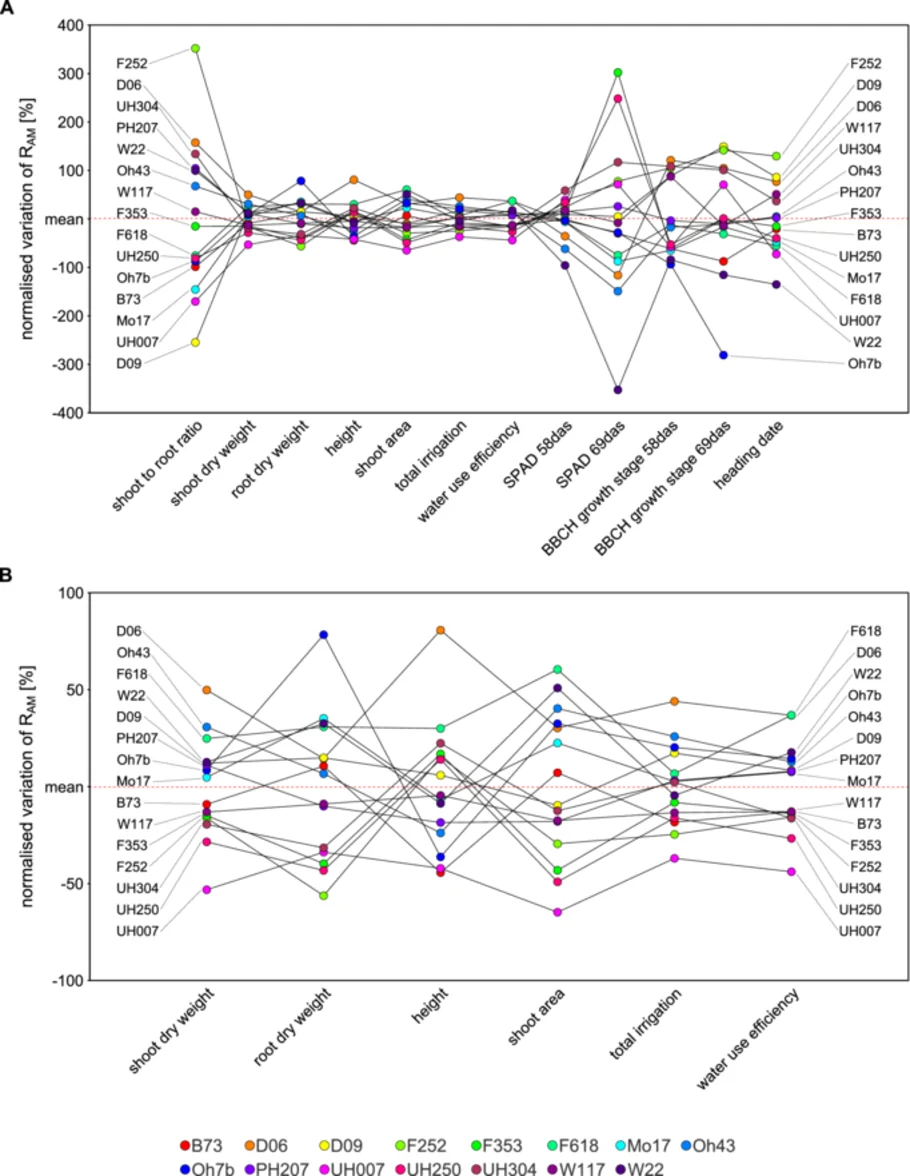
R_AM_ across genotypes varies among traits. The deviation of each genotype's R_AM_ from the mean R_AM_ across all genotypes, expressed as a percentage, is shown for each trait. R_AM_ for each genotype are represented by circles, colour‐coded and connected by lines for easier identification. The mean R_AM_ is indicated by a red dotted line. (A) All traits and (B) a subset to zoom in on traits with lower variation. Data are from experiment 1. [Color figure can be viewed at wileyonlinelibrary.com]

To further test whether R_AM_ of one trait could be inferred from R_AM_ of other traits, we performed an exploratory ridge‐regression analysis on genotype‐level R_AM_ values using leave‐one‐out cross‐validation (Supporting Information S2: Table S5). Predictability was strongly trait‐dependent. Within the growth‐related traits, R_AM_ of shoot surface area was predicted particularly well by the remaining growth traits (*R* ^2^ = 0.781, FDR < 0.001), as were R_AM_ of root dry weight (*R* ^2^ = 0.552, FDR = 0.021), R_AM_ of shoot dry weight (*R* ^2^ = 0.534, FDR = 0.021). R_AM_ of SPAD was also significantly, but more moderately, predicted by the remaining growth traits (*R* ^2^ = 0.392, FDR = 0.031). In contrast, R_AM_s of many ionomic traits (see below) showed weak or no predictability from R_AM_s of other ionomic traits. Prediction of R_AM_ across all traits was detectable for a subset of traits, for example, for R_AM_ of P concentration from growth traits (*R* ^2^ = 0.421, FDR = 0.021) and R_AM_ of shoot surface area from ionomic traits (*R* ^2^ = 0.199, FDR = 0.011), but was generally weaker than prediction within the growth traits. These results indicate that R_AM_ is not regulated by a single uniform response pattern across all traits, but instead shows selective coordination among subsets of traits, suggesting that different genetic loci are involved in shaping different subsets of traits.

### 3.4 AM Plants Exhibit Increased Gross WUE

The automated greenhouse facility used in experiment 1 allowed precise control over the watering of each pot, enabling the collection of detailed data on water usage for each plant. As expected, due to stronger growth, AM plants overall consumed more water than control plants (Supporting Information S2: Figure S10A), leading to a strong positive correlation between shoot dry weight and the amount of irrigated water (Supporting Information S2: Figure S10B). Despite the higher water consumption AM plants exhibited significantly increased gross WUE (which includes evaporation from pots) compared to control plants (Supporting Information S2: Figure S10C), while the water content calculated by subtracting the dry weight from the fresh weight (Figure 1C,E, Supporting Information S2: Figure S11A,C) of AM plants was very similar between AM and control plants (Supporting Information S2: Figure S11B,D). These findings suggest that AM not only enhances plant growth and thus water usage but also the efficiency of water use.

### 3.5 AM Association Increases Uptake of All Elements, Especially Phosphate and Zinc

To understand to which extent AM impacts uptake of nutrients and other elements in the maize lines, we analyzed the concentration of 23 elements (As, B, Ca, Cd, Cr, Cs, Cu, Fe, K, Li, Mg, Mn, Mo, Na, Ni, P, Pb, Pt, Rb, S, Sr, V, Zn) in shoots of individual plants by ICP‐MS. The shoot concentration of the important nutrients P and Zn increased in response to AM in all lines to variable extents, except for F353, which displayed the highest concentration in shoot zinc in control plants and a strong drop in zinc concentration upon AM (Supporting Information S2: Figure S12, left column). This confirms overall the positive effect of AM on P and Zn nutrition (Sawers et al. 2017; Yu et al. 2024), but also highlights its dependence on the plant genotype. The shoot concentrations of Cr, Cu, Li, Ni, Pb and V remained approximately the same between control and AM plants except for UH304 and W117, showing an increase in Li and Mo17, D06, D09 and B73, showing a strong increase in Pb, which may be important when breeding for reduced heavy metal uptake (Supporting Information S2: Figure S12, left column). The shoot concentration of all other elements (As, B, Ca, Cd, Fe, K, Mg, Mn, Mo, N, Rb, S, Sr) decreased upon AM, which is a common effect of AM attributed to dilution of ions due to the increase in shoot growth. Exceptions were the lines B73 and Mo17, which showed a strong AM‐induced increase of B and Mo17, which showed an increase in Fe, suggesting that these lines are particularly efficient in B and Fe uptake, respectively, either directly through AM fungi or stimulated by AM.

To get insights into quantitative nutrient uptake into the shoot, we calculated the approximate total shoot element content by multiplying the concentration by the determined shoot dry weights (Supporting Information S2: Figure S12, right column). The total shoot content of all elements increased upon AM, confirming that the concentration decrease of many elements upon AM was caused by dilution, with Li in the lines UH304 and W117 being one exception (Supporting Information S2: Figure S12, right column). For most elements, there were considerable variations among maize lines in their ability to profit from AM, with W22 profiting particularly strongly for N and K shoot content, Mo17 and Oh43 profiting strongly for Zn shoot content and Mo17 and D06 for Fe shoot content (Supporting Information S2: Figure S12, right column).

To investigate co‐variance between elements (Ramirez‐Flores et al. 2017), we performed a principal component analysis (PCA) for element concentration and for element shoot content (Figure 3A,B). For both parameters, there was a clear separation of control and AM plants along PC1, explaining 76% of the variation for shoot element concentration and 29% for shoot content, and there was little overlap in confidence intervals. Further, the PCA reflects the intuitive evaluation of shoot element concentrations (Supporting Information S2: Figure S12) with most elements being present in higher concentrations in shoots of control plants than of AM plants (Figure 3A). P (together with Cs) also clearly contributes to PC1 but points in the opposite direction with respect to most elements, reflecting the increase in P shoot concentration in AM plants. Also, Na, Ni, Pt, Pb and Zn point towards the direction of AM plants along PC1, but show a stronger contribution to PC2 (Figure 3A). Interestingly, Ramirez‐Flores et al. (2017) found similar patterns in a collection of maize lines representing the American breeding pool for the leaf concentration of P and Ni, pointing in the direction of AM plants, and for Ca, Mg, Mn, Mo and Sr, pointing in the direction of control plants. Only Zn pointed towards control plants in their experiment, while Zn concentration increased in AM maize shoots in our experiment. It is possible that overall, the maize lines in our experiment have a stronger ability to profit from fungus‐delivered Zn, or the fungus used in their experiment (*Funnelliformis mossae*) is less efficient in providing Zn as compared to *R. irregularis*. Alternatively, the difference could be caused by variations in Zn availability between the respective substrates.

**Figure 3:**
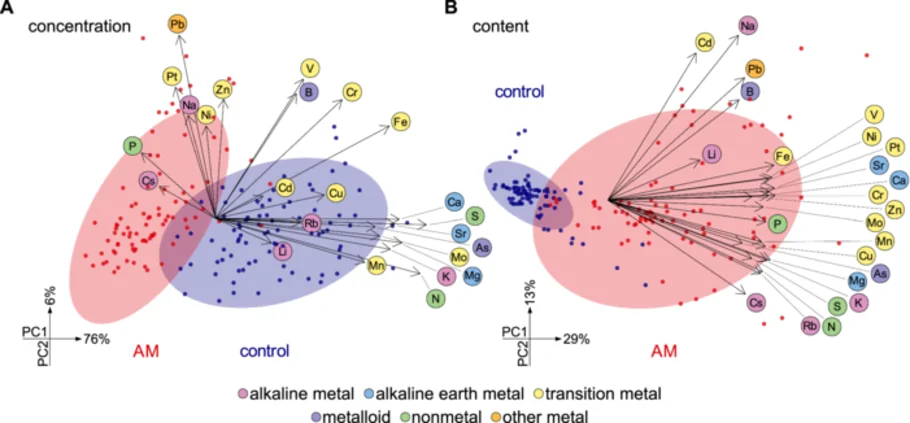
AM symbiosis impacts the shoot concentration and content of 24 elements. Principal component analyses (PCA) were conducted on datasets from all 15 genotypes grown in experiment 1. Data from AM (red) and control (blue) plants are colour‐coded. The 95% confidence intervals are represented as ellipses, coloured according to the treatment. (A) PCA based on the concentration of elements and (B) based on the shoot content of elements. The plot displays the first two principal components (PC; PC1 = *x*‐axis, PC2 = *y*‐axis). The direction and length of the arrows indicate the contribution of each selected element to the principal components. [Color figure can be viewed at wileyonlinelibrary.com]

The increase in shoot content of all elements in AM plants is clearly reflected in their contribution to PC1 (Figure 3B). However, the elements separate into three groups. One large group including Ca, Cr, Fe, Mo, Ni, P, Pt, Sr, V and Zn contributes almost exclusively to PC1 and thereby explains most of the variation, while another group consisting of B, Cd, Na, Pd, Li contributes almost equally to both PCs with a stronger tendency towards PC2 and another group containing As, Cs, Cu, K, Mg, Mn, N, Rb, S contributes approximately equally to both PCs but in the opposite direction (Figure 3B). Thus, there seems to be coordination in the uptake of some element groups.

### 3.6 Correlations Among Nutrient Concentrations Shift Between Control and AM Plants

To understand whether the concentration or shoot content may explain differences in plant growth, we pooled data from all genotypes and performed a correlation analysis with all elements against root and shoot dry weight and shoot area for control and AM plants separately. For AM plants, we also included root length colonization with fungal structures (intraradical hyphae, arbuscules and vesicles) into the analysis. We focused mainly on phosphorus as this is thought to be the most important element provided by AM fungi to plants (Chiu and Paszkowski 2019). In control plants, P concentration (along with K, Cs and Rb) was strongly positively correlated with plant growth (shoot dry weight, root dry weight, shoot area), whereas in AM plants, a strong negative correlation was observed along with S, N, Ca, Cu, Mn, Mo and Sr (Figure 4A,B). This was surprising as P concentration is increased in AM plants, while the concentration of the other elements named here is overall decreased or the same in AM as compared to control plants (Supporting Information S2: Figure S12). This indicates that the increased R_AM_ may cause a dilution of this essential element, although the concentration in general was significantly higher in AM than in control plants. Interestingly, the strong correlation of shoot phosphorus with Cs, Rb, K, and As in control plants shifted to a strong correlation with Ca, Mg, Mn, S, Cu, and Fe in AM plants, while N and Mo were strongly correlated with P in both conditions (Figure 4A,B). This suggests that a potential link in the uptake routes of a group of nutrients changes between control and AM plants. The correlation between arbuscule abundance and phosphorus concentration was not significant, as previously observed (Sawers et al. 2017). However, arbuscule abundance was negatively correlated with the concentration of elements such as Pt, As, B, Pb and V.

**Figure 4:**
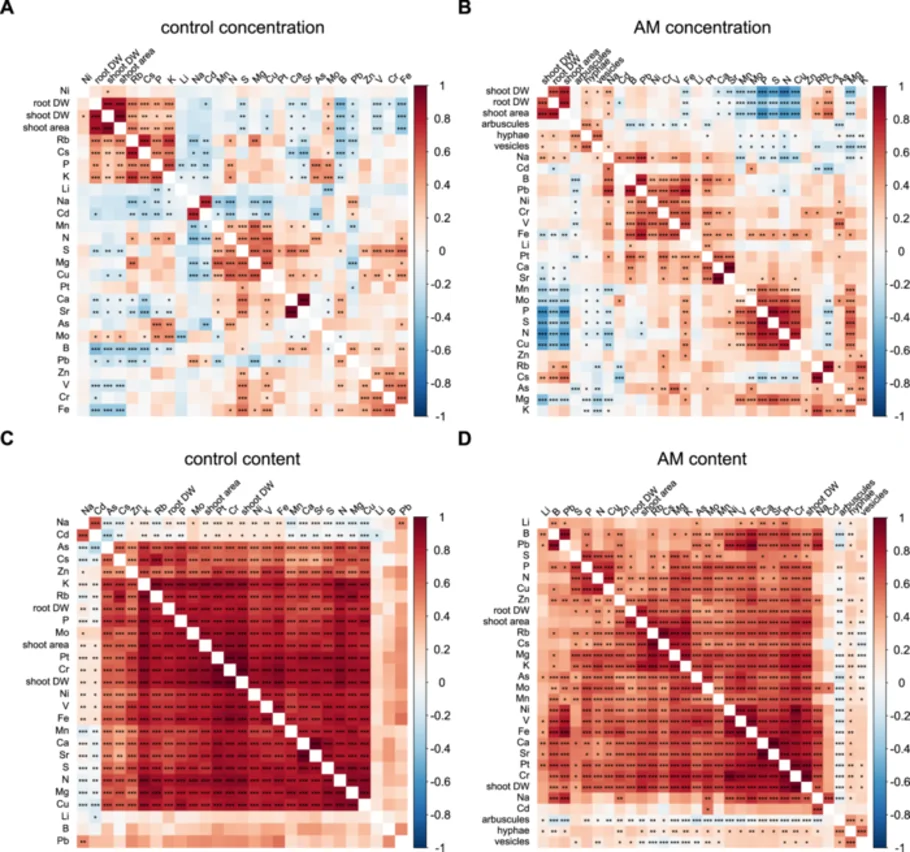
Correlation matrix of selected traits and elemental content/concentrations in maize shoot tissues. Correlations for the (A, B) concentration and the (C, D) total content of selected elements in plant shoots. Separate matrices for (A, C) control and (B, D) AM plants are shown. In addition to element quantities, shoot area, shoot dry weight (DW), and root DW were included for both control and AM plants. For AM plants, colonization traits were also analyzed. Asterisks denote the significance levels of the correlations (*n* = 4–7, *t*‐test, *p < 0.05, **p < 0.01, ***p < 0.001). Pearson correlation coefficients (*r*) are visually represented by a colour gradient, ranging from blue for negative correlations to red for positive correlations. The matrices are ordered using hierarchical clustering. All data are from experiment 1. [Color figure can be viewed at wileyonlinelibrary.com]

We also correlated the total shoot content of all elements with plant growth parameters and root length colonization with fungal structures. Overall, the correlations among the shoot contents of most elements and plant growth parameters were very strong and stronger for control plants than for AM plants (Figure 4C,D). Exceptions were Cd in AM plants and Na, Cd, Li, B, and Pb in control plants. This indicates that the overall strong correlation between shoot element content and growth parameters cannot exclusively be explained by how the total shoot element content was derived. Moreover, a strong positive correlation was found between the total shoot content of Na and Cd in both AM and control plants (Figure 4C,D).

Root colonization by arbuscules was negatively correlated with the total shoot content of heavy metals such as lead and arsenic (Figure 4D). A previous study has shown that reduced AM colonization in maize increases lead and manganese concentrations while simultaneously decreasing zinc and copper concentrations in leaves (Hovsepyan and Greipsson 2004). In another study, mitigation by AM of heavy metal uptake into the shoot was strongly genotype dependent, with, however, more genotypes reducing heavy metal uptake in the presence of AM (Yin et al. 2021). Our data are consistent with AM‐mediated changes in metal partitioning or translocation.

### 3.7 Variation in Direct and AM‐Mediated Phosphate, Nitrate and Sulphate Uptake Among Maize Lines

In addition to the total elements, we also quantified the soluble ions of three main nutrients, namely phosphate, nitrate and sulphate, by ion chromatography and compared them to the total concentration and amount of P, N and S across maize genotypes. As for the element P, also phosphate concentration increased in AM plants, and the magnitude of the increase differed across lines (Figure 5A,B).

**Figure 5:**
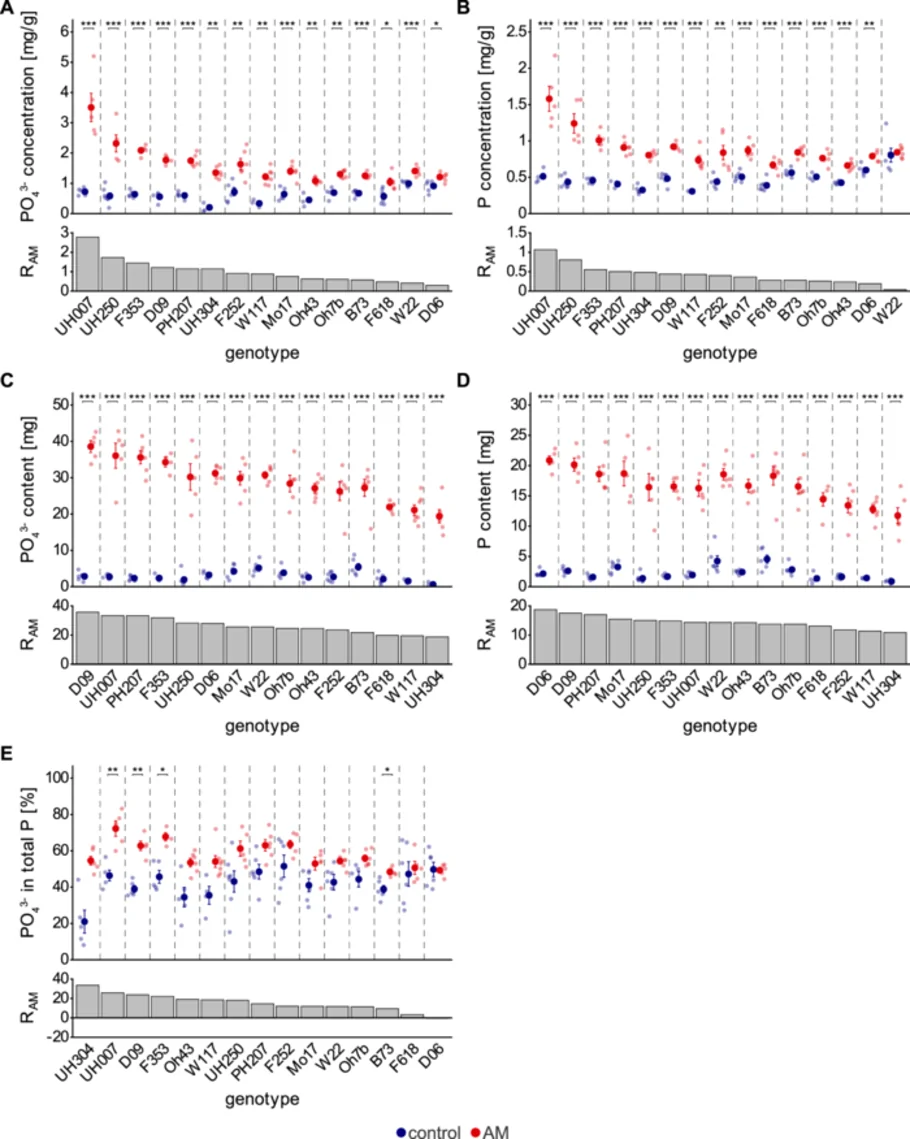
AM‐mediated shoot phosphorus increase varies among 15 maize genotypes. Plants were grown either with (AM; red) or without (control; blue) the arbuscular mycorrhiza fungus *Rhizophagus irregularis*. (A) Shoot phosphate concentration, (B) shoot phosphorus concentration, (C) shoot phosphate content, (D) shoot phosphorus content and (E) the proportion of phosphorus present as phosphate in total phosphorus measured at 69 das. The upper panel shows values from individual plants (light‐coloured dots) along with the means (dark‐coloured dots) and standard error of the mean for each genotype and treatment combination. Asterisks denote significant differences between treatment means within a genotype (*n* = 4–7, *t*‐test, *p < 0.05, **p < 0.01, ***p < 0.001). The lower panel shows genotype‐specific R_AM_, calculated as the difference between the means of AM plants and control plants. In all panels, genotypes are displayed in decreasing order of R_AM_. Data are from experiment 1. [Color figure can be viewed at wileyonlinelibrary.com]

In contrast, just as for total nitrogen and sulphur, also the concentrations of sulphate were lower in AM plants compared to control plants (Figures 6A,B and 7A,B). Notably, nitrate was either undetectable or present in very small concentrations in AM plants, resulting in a strong negative R_AM_ for nitrate across all genotypes (Figure 6A). High nitrate concentrations above 0.6 mM have been shown to inhibit maize growth, leading instead to the accumulation of nitrate in roots and shoots (Khamis and Lamaze 1990). We speculate that control plants, lacking AM association, were unable to reach their full growth potential and, as a result, began accumulating excess nitrate. In contrast, undetectable shoot nitrate in AM plants is consistent with altered nitrate uptake or assimilation; however, alternative explanations such as partitioning, dilution or detection limits cannot be excluded (Figure 6A). In addition, the concentration of nitrate and sulphate in shoots of mock plants differed more substantially among lines than for phosphate (Figures 5A, 6A and 7A). Likely, these two nutrients were less limiting in the substrate than phosphate, which is strongly retained by clay (Edzwald et al. 1976), such that genetic polymorphisms affecting direct nitrate and sulphate uptake could take effect.

**Figure 6:**
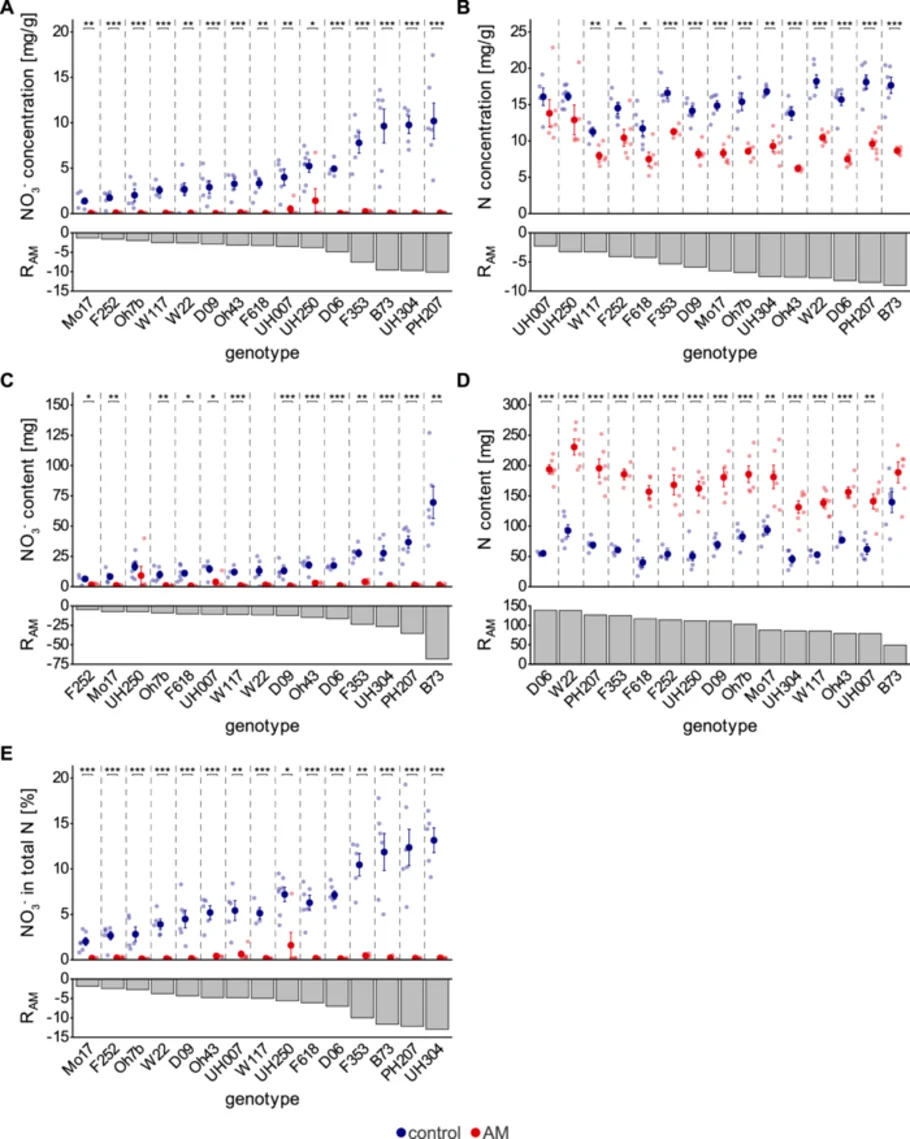
Nitrate content decreases and nitrogen increases in response to AM in 15 maize genotypes. Plants were grown either with (AM; red) or without (control; blue) the arbuscular mycorrhiza fungus *Rhizophagus irregularis*. (A) Shoot nitrate concentration, (B) shoot nitrogen concentration, (C) shoot nitrate content, (D) shoot nitrogen content and (E) the proportion of nitrogen present as nitrate in total nitrogen measured at 69 das. The upper panel shows values from individual plants (light‐coloured dots) along with the means (dark‐coloured dots) and standard error of the mean for each genotype and treatment combination. Asterisks denote significant differences between treatment means within a genotype (*n* = 4–7, *t*‐test, *p < 0.05, **p < 0.01, ***p < 0.001). The lower panel shows genotype‐specific R_AM_, calculated as the difference between the means of AM plants and control plants. In all panels, genotypes are displayed in decreasing order of R_AM_. Data are from experiment 1. [Color figure can be viewed at wileyonlinelibrary.com]

**Figure 7:**
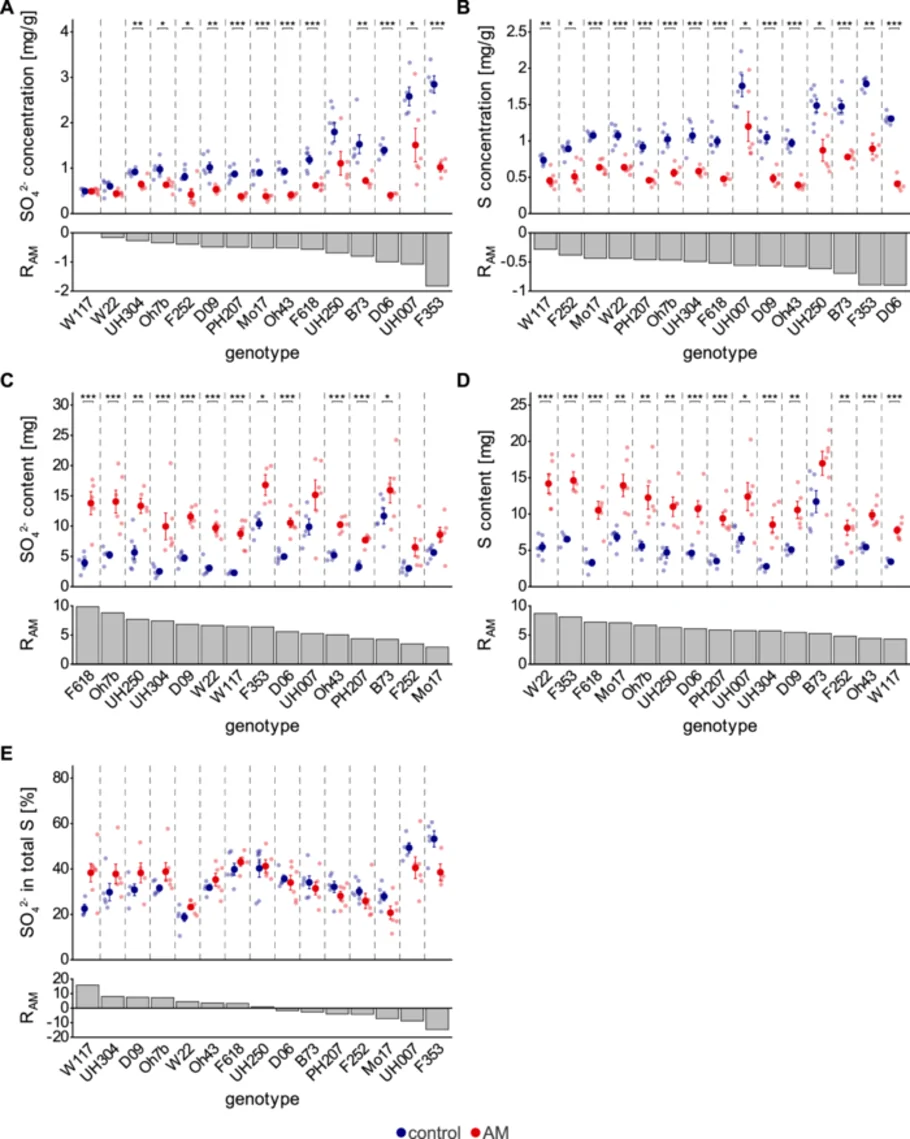
AM‐mediated changes in shoot sulphur concentration and content vary among 15 maize genotypes. Plants were grown either with (AM; red) or without (control; blue) the arbuscular mycorrhiza fungus *Rhizophagus irregularis*. (A) Shoot sulphate concentration, (B) shoot sulphur concentration, (C) shoot sulphate content, (D) shoot sulphur content, and (E) the proportion of sulphur present as sulphate in total sulphur measured at 69 das. The upper panel shows values from individual plants (light‐coloured dots) along with the means (dark‐coloured dots) and standard error of the mean for each genotype and treatment combination. Asterisks denote significant differences between treatment means within a genotype (*n* = 4–7, *t*‐test; *p < 0.05, **p < 0.01, ***p < 0.001). The lower panel shows genotype‐specific R_AM_, calculated as the difference between the means of AM plants and control plants. In all panels, genotypes are displayed in decreasing order of R_AM_. Data are from experiment 1. [Color figure can be viewed at wileyonlinelibrary.com]

As already described for total P, N and S, also the total amount of phosphate and sulphate was higher in AM plants than in control plants (Figures 5C,D, 6C,D and 7C,D). This underscores the enhanced nutrient uptake through AM, while the lower concentrations of nitrogen, sulphate, and sulphur in shoots of AM plants likely result from dilution through enhanced plant growth. The strong increase of N in AM plants may be explained by assimilation of all available nitrate into other molecules and/or potentially additional N‐uptake via ammonium (Hui et al. 2022). We calculated the percentage of P, N and S present as phosphate, nitrate or sulphate. This revealed that eight lines accumulated significantly enhanced levels of storage phosphate in the presence of AM as compared to control plants, whereas for all other lines the percent P present as soluble phosphate was not or only insignificantly enhanced (Figure 5E). As mentioned above, free nitrate levels differed significantly among lines in mock conditions (Figure 6E). The same was true for sulphate (Figure 7E), and no statistically significant difference was found in AM plants, indicating that the increase in sulphate uptake in AM plants does not lead to increased storage pools, likely due to increased assimilation of S into other molecules. In summary, we found variation in direct and mycorrhizal phosphate, nitrate, and sulphate uptake, as well as in their assimilation into other molecules, among the 15 maize lines.

## 4 Discussion

Here, we characterized the impact of AM with the fungus *R. irregularis* on the growth and nutrient uptake of 15 maize inbred lines with 9 parental lines from the Dent and 1 from the Flint EU‐NAM population, as well as 5 parental lines from the US American Dent breeding pool (Bauer et al. 2013; McMullen et al. 2009). We used an automated LemnaTec growth system (experiment 1, Figure 1, Supporting Information S2: Figure S1), enabling non‐destructive shoot surface recordings over time. In this system, controlled growth conditions and randomization allowed for high reproducibility among replicates and revealed significant variation in growth dynamics among the maize lines in response to AM, visible as growth curves with line‐specific slopes and saturation points. We could confirm the differences in growth dynamics in response to AM for two contrasting lines by using a LemnaTec system in a different location (experiment 2, Supporting Information S2: Figure S3). However, in experiment 2, lack of randomization led to increased variability among replicates, highlighting the benefits of randomization.

xWe found a strong correlation between end‐point shoot dry weight and automatically determined shoot area, confirming the accuracy and efficiency of phenotyping platforms for maize growth measurements (Zhang et al. 2017). The use of a low phosphate‐containing substrate with alkaline pH 7.6, in combination with slow‐release fertilizer without phosphate, caused severe starvation symptoms and stunted growth in control plants but revealed the full potential of AM to increase plant growth (Video S1). In fact, we observed an impressive increase in shoot biomass in response to AM that varied between 165% and 633% among the 15 lines. The variation in shoot dry weight among the 15 maize lines was not correlated with endpoint root colonization levels (Supporting Information S2: Figure S5), which aligns with previous findings in American maize inbred line collections (Kaeppler et al. 2000; Sawers et al. 2017). This suggests that it may not be the colonization level but the efficiency of AM‐mediated nutrient uptake, for example, through differences in the development of extraradical fungal hyphae (Sawers et al. 2017) that determines variation in R_AM_ of growth. However, it remains unclear whether colonization dynamics at earlier time‐points may influence the plant growth outcome. Experiment 3, in which we recorded a time course of root colonization along with root and shoot dry weight and green leaf area, demonstrated that R_AM_ increases in parallel with colonization levels for the three traits (Supporting Information S2: Figures S6 and S7). It remains to be determined whether increased colonization drives enhanced plant growth or whether increased plant growth facilitates stronger root colonization. It will be interesting in future studies to unravel the dynamic interactions between plant growth, root colonization and extraradical mycelium development over time, especially under varying environmental conditions.

As AM plants accumulated about 1.65‐ to 6.33‐times more biomass than control plants, their gross water use was significantly increased, and the amount of irrigation positively correlated with shoot dry weight (Supporting Information S2: Figure S10). Nevertheless, we observed that AM significantly increased WUE across all lines, as previously reported for rice (Watts‐Williams et al. 2025). This may be due to AM‐mediated increase in root system size and additional exploration of the substrate by hyphae, leading to better usage of moisture across the pot that may otherwise evaporate (Abdalla et al. 2023).

As we recorded R_AM_ for several traits, we observed that the maize lines changed position in the R_AM_ ranking depending on the trait, indicating that the R_AM_ is strongly trait‐specific rather than uniform across traits. For example, Oh7b showed a strong positive deviation from the mean for R_AM_ of root dry weight across all genotypes and a negative deviation for R_AM_ of height, while Oh43 performed well for many traits, especially shoot dry weight, confirming an earlier report from Sawers et al. (2017) showing that in this line, high shoot R_AM_ was correlated with strong proliferation of the fungal extraradical mycelium. In contrast, the Flint line UH007 showed low R_AM_ for most traits (Figure 2). An important next step will be to reveal the QTLs and genetic polymorphisms underlying the R_AM_ variation for the traits with the largest potential for agricultural benefits.

We determined the concentration and calculated the total amount of 24 elements and three nutrient ions (phosphate, nitrate and sulphate) in shoots of the 15 maize lines when colonized by *R. irregularis* or grown as non‐colonized controls. Similar to Ramirez‐Flores et al. (2017), we observed coordinated changes of groups of elements upon AM (Figure 3), although the groupings partially differed from the observations made by Ramirez‐Flores et al. (2017). It will be interesting to determine whether coordinated changes of certain elements are caused by the specific substrate, the maize genotypes or potentially by the fungal species, as Ramirez‐Flores et al. (2017) used *F. mossae*, while we employed *R. irregularis* in our experiments.

We observed large genotypic variation in AM‐mediated nutrient acquisition, and this may potentially be another factor driving R_AM_ differences. However, as for growth traits, we observed that AM‐mediated increases in shoot element content were strongly trait‐specific and not uniformly predictable across elements. The concentration of most elements and ions, except phosphate, decreased in shoots of AM plants most likely due to dilution by increased growth, as the total shoot content of all elements increased upon AM with variation among maize lines (Figures 5, 6, 7, Supporting Information S2: Figure S12). However, the shoot concentration of some elements, for example B, Zn, Fe and Pb, increased upon AM in some lines (Supporting Information S2: Figure S12). Especially, Mo17 displayed an increase in concentration of these ions upon AM, indicating that uptake outweighed dilution caused by increased growth. The concentration of these ions was strongly correlated in AM plants (Figure 4), suggesting that increased AM‐mediated uptake of elements that improve plant nutritional value, such as Fe, is potentially genetically coupled to increased uptake of toxic elements such as Pb. Interestingly, Oh43 showed a strong AM‐mediated increase in shoot Zn concentration without an increase in Pb concentration. This observation points towards opportunities to genetically dissect genetic loci for AM‐improved Zn uptake and biofortification of crops from those of detrimental AM‐improved Pb uptake.

Overall, we observed a negative correlation between arbuscule abundance and the accumulation and concentration of toxic elements such as Cr, Ni, As and Pb (Figure 4). This supports earlier work by Dehn and Schüepp (1990) and Hovsepyan and Greipsson (2004), which showed that AM fungi can reduce the uptake of heavy metals. Interestingly, while As and P concentrations were positively correlated in shoots of control plants, this correlation was lost in AM plants (Figure 4) and As concentrations decreased in all AM plants, while P and phosphate concentrations increased in all AM plants despite the likely dilution of element concentration due to increased shoot growth (Supporting Information S2: Figure S12, Figure 5A,B). Arsenate is taken up by plant phosphate transporters of the Pht1 family and is toxic to plants, animals and humans (Zhao et al. 2009). It has been previously described that AM increases the P:As uptake ratio of barley and maize (Christophersen et al. 2009; Zhou et al. 2023), and this can likely be explained by an AM‐mediated decrease in expression of plant Pht1 transporters localized in the root epidermis and a switch to the mycorrhizal phosphate uptake pathway (Christophersen et al. 2009; Sawers et al. 2008). However, this means that phosphate importers in AMF extraradical hyphae must be more selective for phosphate than plant Pht1 transporters, thereby discriminating better against arsenate. Alternatively, AMF may accumulate arsenate in their extraradical hyphae without providing all acquired arsenate to plants, or AM may induce a mechanism that prevents transport of As from roots to shoots (Bai et al. 2008; Sudová and Vosátka 2007; Zhan et al. 2018).

The AM‐mediated increase in phosphate uptake in soils with poor phosphate availability is one major driver of AM‐mediated plant growth (Smith et al. 2011). However, in our study, genotypes with high R_AM_ for phosphate concentration, phosphate shoot content and phosphorus concentration tended to exhibit lower biomass gains. This suggests that while these genotypes receive more phosphate from the fungus, they may have to provide more carbon to support the fungal partner for extraradical hyphal growth (Kiers et al. 2011), indicating a potential tradeoff between nutrient acquisition and carbon allocation. Alternatively, their genetic makeup may not allow them to sufficiently convert increases in phosphorus into growth. This may also explain why we observed a positive correlation between phosphorous and shoot and root dry weight in control plants but a negative correlation of these traits in AM plants, although R_AM_ was positive across all genotypes for phosphate and phosphorus shoot concentration and content (Figures 4 and 5).

AM clearly promoted phosphate uptake more strongly than nitrate and sulphate uptake, as the shoot concentrations of nitrate, nitrogen, sulphate and sulphur decreased in shoots of AM plants as compared to control plants. Additionally, we observed low to undetectable nitrate concentrations and shoot content in AM plants. This may indicate that AM plants rapidly assimilate nitrate into other molecules or that more nitrate is partitioned in roots as compared to shoots. Further, in our experiment, the plants likely strongly relied on nitrogen supplied by the fungus, and most of this may have been delivered in the form of ammonium, which appears to be more readily transferred to maize than nitrate, at least by the AMF *Glomus aggregatum* (Tanaka and Yano 2005).

Application of AM in agricultural systems holds promise to stabilize yield under decreased fertilizer application. However, its successful application requires the right combination of plant and fungal genotypes with environmental and soil conditions (Berger and Gutjahr 2021; Lutz et al. 2023; Rog et al. 2025). Variation in R_AM_ among genotypes of the same crop species provides opportunities for breeding AM‐optimized crops, but it remains to be seen whether this can be successfully put into practice and applied to different environments. As different growth‐related AM‐responsive traits and uptake of different elements were not uniformly coordinated, careful selection of the desired AM‐enhanced trait is required. One promising opportunity lies in the genetic dissection of AM‐increased uptake of nutritious ions from the uptake of toxic heavy metals. In this study, we used highly controlled conditions and randomization of plants. In the next step R_AM_ of the analyzed lines needs to be determined in the field under different environmental conditions to understand the extent of genotype × environment interaction.

This study was funded by the Bavarian State Ministry of Environment and Consumer Protection (Germany) through the BayKlimaFit project network to C.G. and M.H., by the Federal Ministry of Education and Research (BMBF, Germany) within the scope of the funding initiative ‘Plant Breeding Research of the Bioeconomy’ (Funding ID: 031B0882J; http://www.europeanmaize.net/) to C.G. and by a core grant of the Max Planck Society to C.G. S.K.'s work is supported by the Deutsche Forschungsgemeinschaft (DFG) under Germany's Excellence Strategy – EXC 2048/1 – project 390686111 and within the transregional collaborative research centre TRR 341 ‘Plant Ecological Genetics’, TRR341/1 project 456082119.

## Supporting information


Supporting File 1

Supporting File 2

Supporting File 3

## Data Availability

The data that support the findings of this study are available in the Supporting information of this article.
