# Supplementary material for: Automated Phenotyping Unveils Trait‐Specific Genotypic Variation in Arbuscular Mycorrhiza Responsiveness in Maize (Zea mays)
Source: Plant Cell Environ. 2026 Jul 6;49(11):7957–75. doi: 10.1111/pce.70709 (PMC13646665; doi:10.1111/pce.70709)
Supplement: Supplementary file 1 [file PCE-49-7957-s002.pdf]

# Automated phenotyping unveils trait-specific genotypic variation in arbuscular mycorrhiza responsiveness in maize (*Zea mays*)

Florian Berger[1,2,§], Peter Muth[1,3,§], Wouter K. Vahl[4,A,§], Astrid Junker[5,B], Thomas Altmann[5], Stanislav Kopriva[6], Markus Herz[4], Caroline Gutjahr[1,2,3,*]

[1] Plant Genetics, TUM School of Life Sciences, Technical University of Munich (TUM), Emil Ramann Str. 4, 85354 Freising, Germany

[2] Max-Planck-Institute of Molecular Plant Physiology, Potsdam Science Park, Am Muehlenberg 1, 14476 Potsdam-Golm, Germany

[3] Faculty of Biology, Genetics, LMU Munich, Biocenter Martinsried, Großhaderner Str. 2-4, 82152 Martinsried, Germany

[4] Institute for Crop Science and Plant Breeding, Bavarian State Research Center for Agriculture, Am Gereuth 8, 85354 Freising, Germany

[5] Leibniz Institute of Plant Genetics and Crop Plant Research, OT Gatersleben, Corrensstr. 3, 06466 Seeland, Germany

[7] University of Cologne, Institute for Plant Sciences, Zuelpicher Str. 47b, 50674 Cologne, Germany

§ These authors contributed equally to this work

* Corresponding author: Caroline Gutjahr, gutjahr@mpimp-golm.mpg.de

**Current address**

[A] Naturkundemuseum Ostbayern, Am Prebrunntor 4, 93047 Regensburg, Germany

[B] Syngenta Seeds GmbH, Zum Knipkenbach 20, 32107 Bad Salzuflen

## Supplemental materials

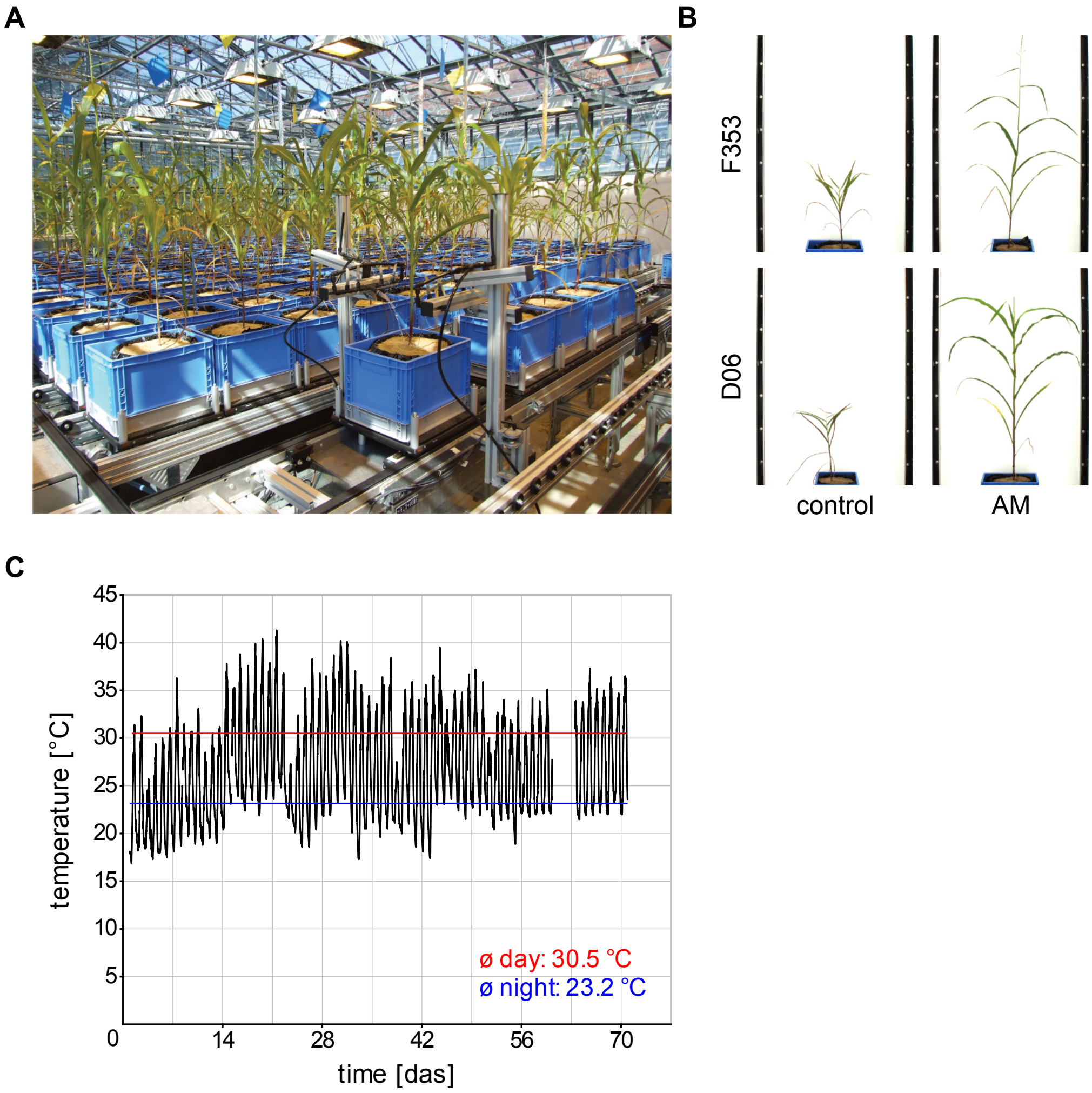


**Supplemental Figure S1.** Experimental setup of experiment 1.
(A) Photo of the LemnaTec system used in experiment 1, with watering station visible in the foreground. (B) Representative images obtained from the LemnaTec-Scanalyzer3D platform, used to calculate shoot surface area. Images depict AM and control plants from genotypes D06 and F353 at 60 days after sowing. (C) Greenhouse temperature profile. Mean daytime (08:00-20:00; red line) and nighttime (20:00-08:00; blue line) temperatures are shown in degrees Celsius (°C) over the course of the experiment.

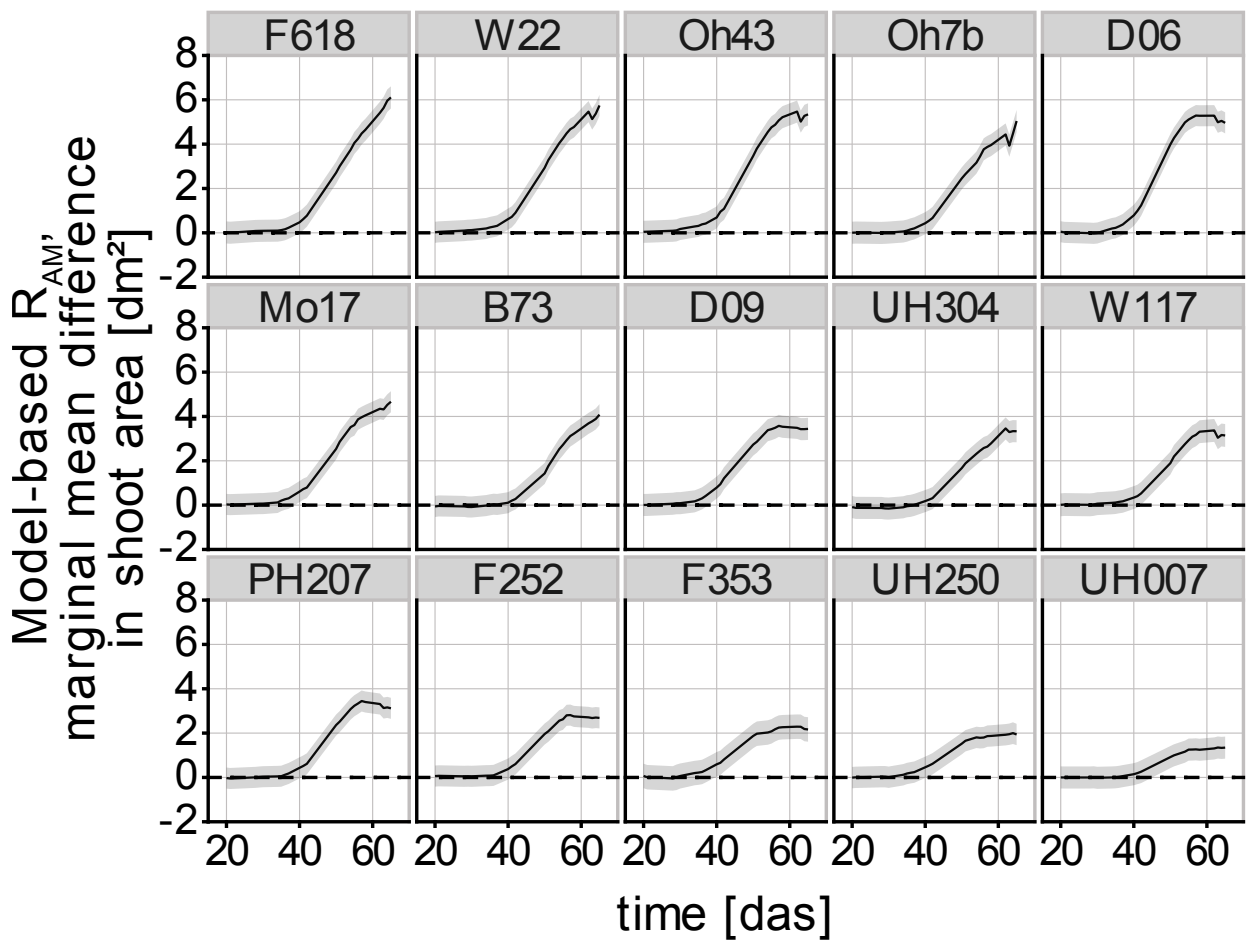


**Supplemental Figure S2.** Model-based AM responsiveness ($R_{AM}$) of shoot area over the course of experiment 1.

$R_{AM}$ was estimated for each genotype and time point using a linear mixed-effects model. $R_{AM}$ was defined as the difference between inoculated (AM) and control plants based on estimated marginal means. Lines represent model-based estimates for each time point, and shaded areas indicate 95% confidence intervals. Measurements were derived from repeated observations of the same plants over time, with plant identity included as a random effect in the model.

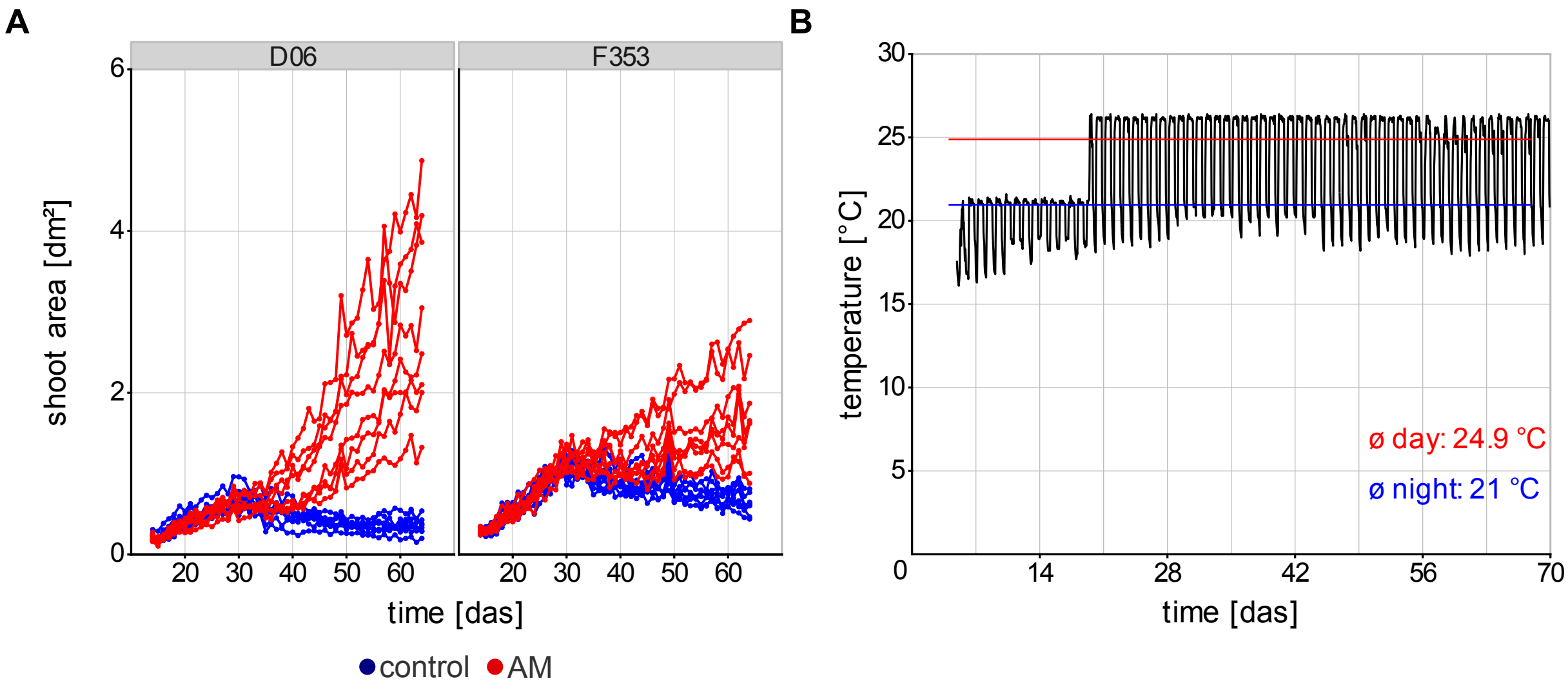


**Supplemental Figure S3.** Independent replication of growth experiment for assessing the growth response to AM of D06 and F353 in experiment 2.

(A) Time-course plots depict shoot area, determined through digital imaging, for two maize genotypes (D06 and F353) grown either with the arbuscular mycorrhiza fungus *R. irregularis* (AM; red lines) or without (control; blue lines). (B) Greenhouse temperature profile (experiment 2). Mean daytime (08:00-20:00; red line) and nighttime (20:00-08:00; blue line) temperatures are shown in degrees Celsius (°C) over the course of the experiment.

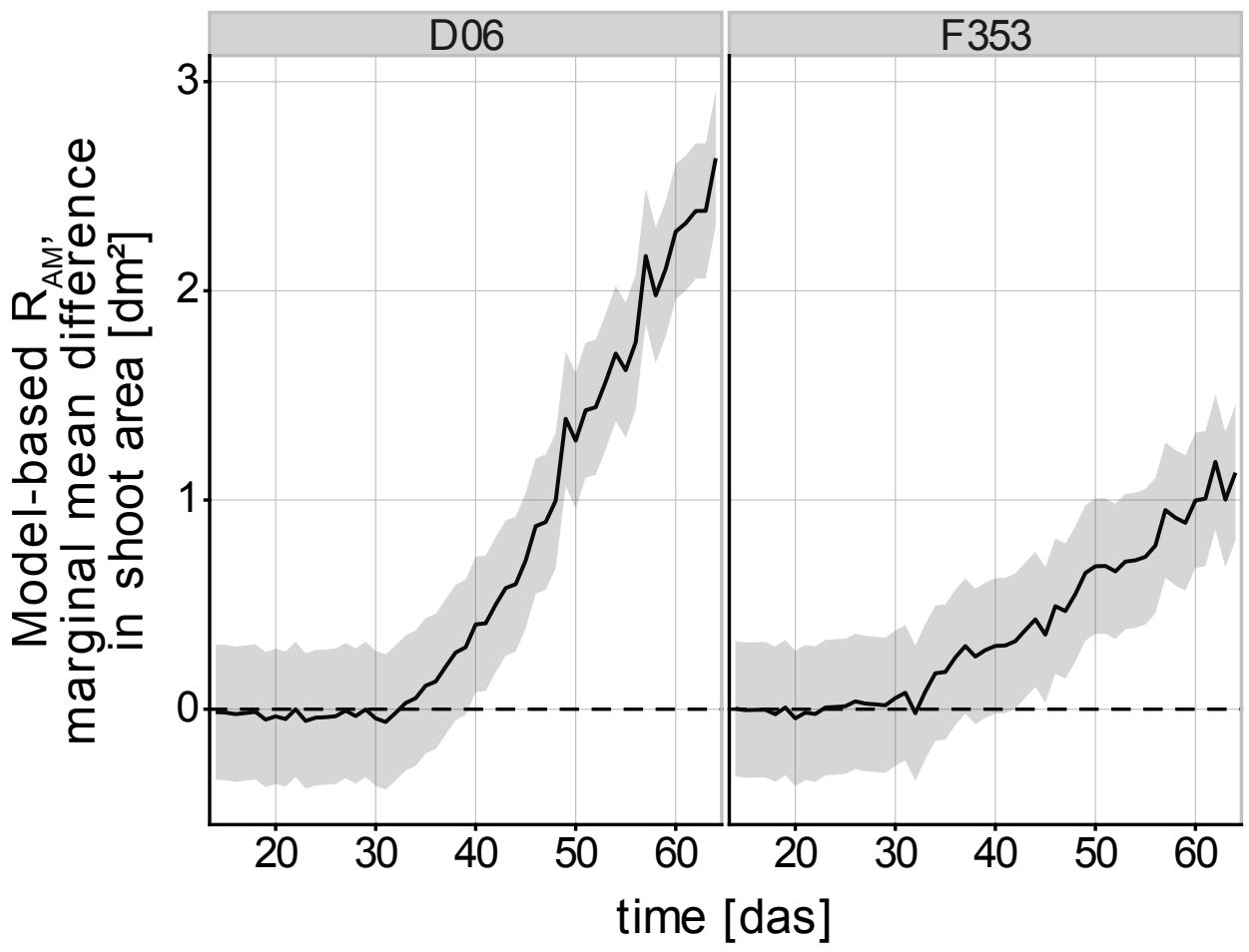


**Supplemental Figure S4.** Model-based AM responsiveness ($R_{AM}$) of shoot area over the course of experiment 2.

$R_{AM}$ was estimated for each genotype and time point using a linear mixed-effects model. $R_{AM}$ was defined as the difference between inoculated (AM) and control plants based on estimated marginal means. Lines represent model-based estimates for each time point, and shaded areas indicate 95% confidence intervals. Measurements were derived from repeated observations of the same plants over time, with plant identity included as a random effect in the model.

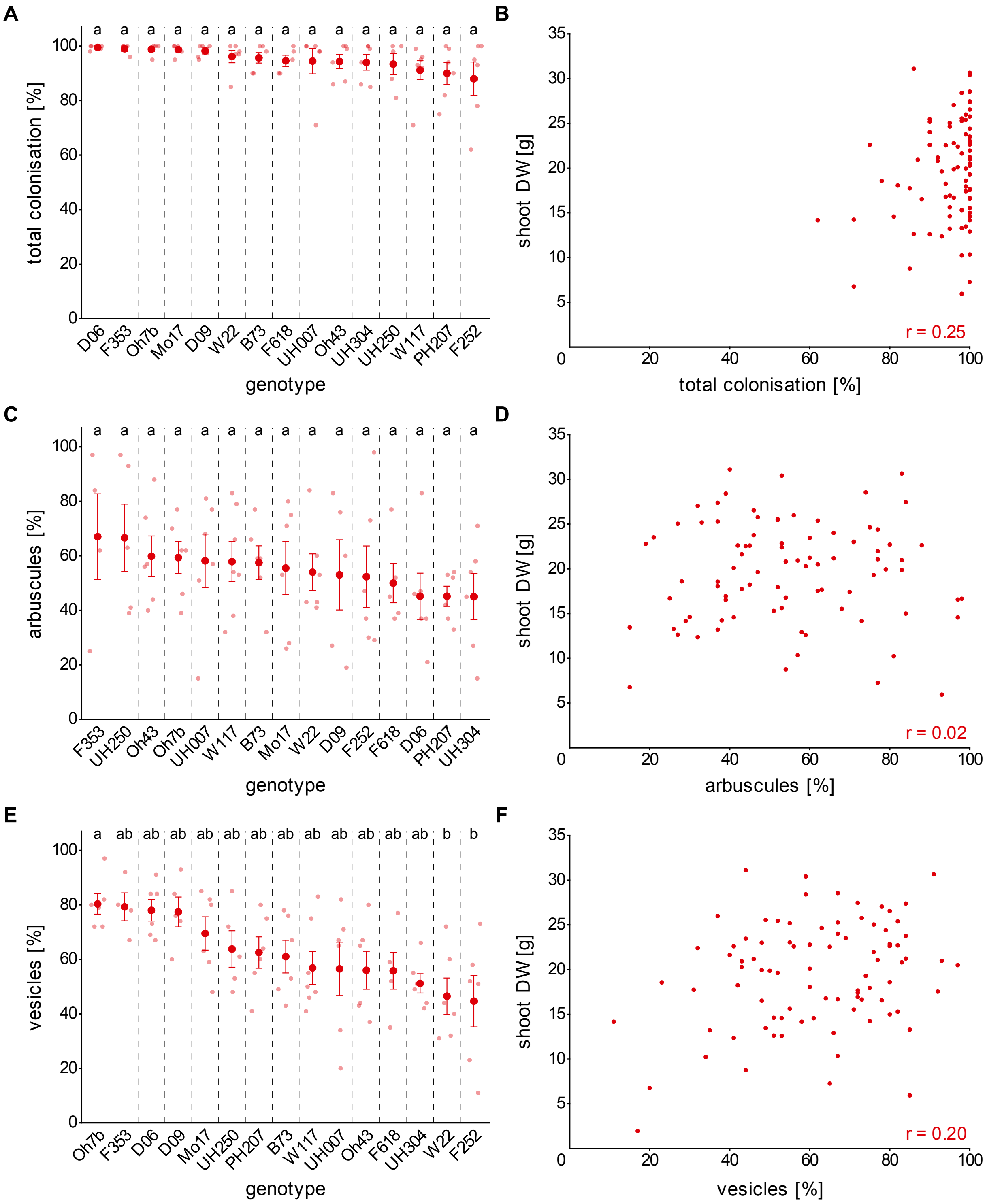


**Supplemental Figure S5.** Variation in root colonization of inoculated plants and its correlation with shoot dry weight (experiment 1).

The percentage of root length colonized by *R. irregularis* is shown for (A) total colonization, (C) arbuscules and (E) vesicles. Genotypes are grouped by letters, where those sharing the same letter do not differ significantly at α = 0.05. The genotypes are displayed in order of decreasing colonization means. Datapoint of individual plants are shown with light-colored dots, means with dark-colored dots and standard errors of the mean by error bars. Shoot dry weight was correlated with the percentage of (B) total root colonization, (D) colonization by arbuscules and (F) vesicles. Pearson correlation coefficients are indicated in red in the lower right corner of each plot (B: r = 0.25, p = 0.021, n = 86, 95% confidence level [0.04, 0.44]; D: r = 0.02, p = 0.889, n = 86, 95% confidence level [-0.20, 0.23]; F: r = 0.20, p = 0.064, n = 86, 95% confidence level [-0.01, 0.40]).

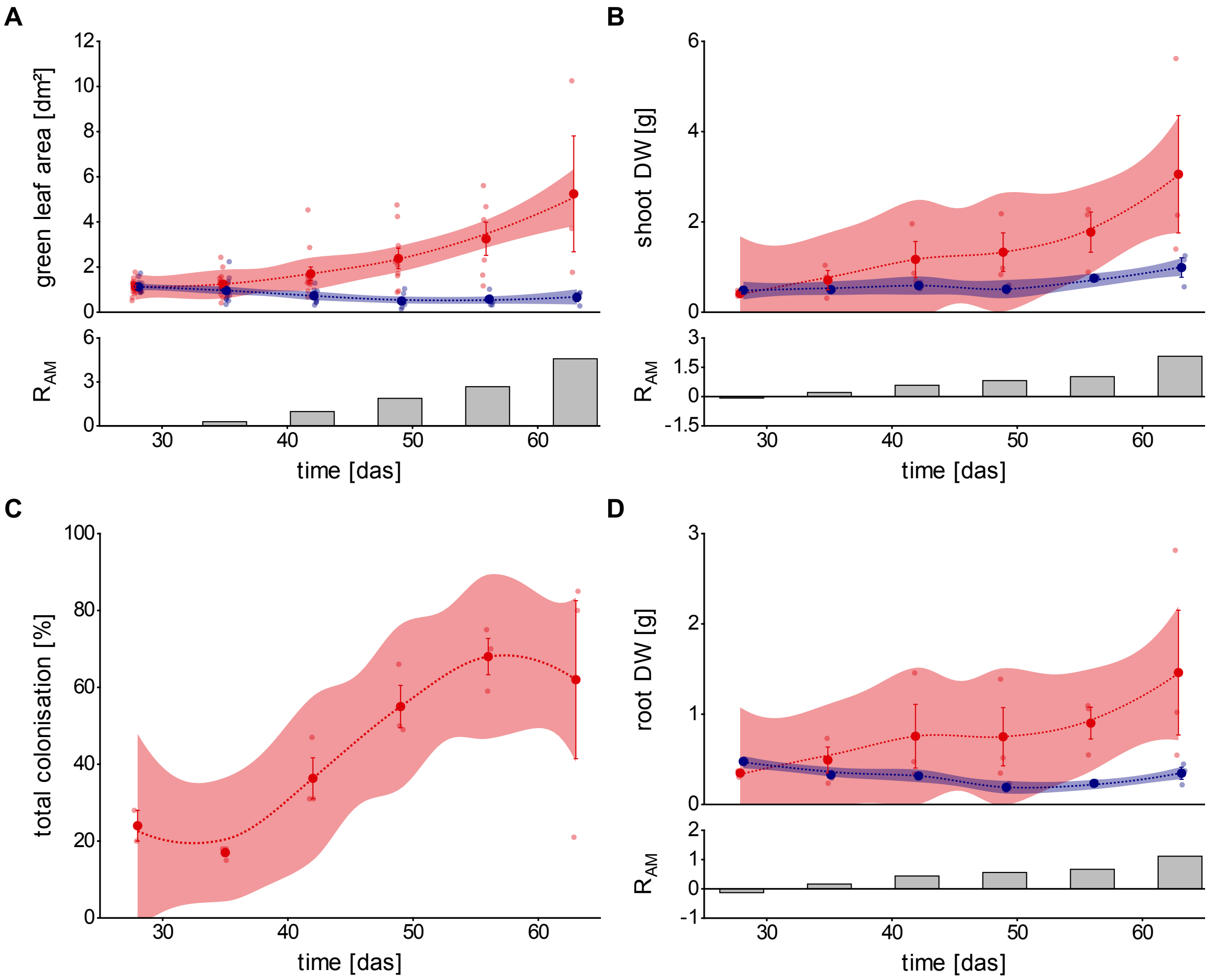


**Supplemental Figure S6.** Time course of the AM effect on three growth traits of B73 (experiment 3).

(A) Green leaf area, (B) shoot dry weight, (C) total root length colonization and (D) root dry weight were measured at 7-day intervals starting from 28 days after sowing (das). For each treatment, smoothed trend lines represent the fitted values, with shaded areas indicating the 95% confidence intervals. In panels (A), (B) and (D) the upper panel shows individual plants data points (light-colored dots) as well as the mean values (dark-colored dots) and the standard errors of the mean for each treatment. The lower panel displays the AM response.

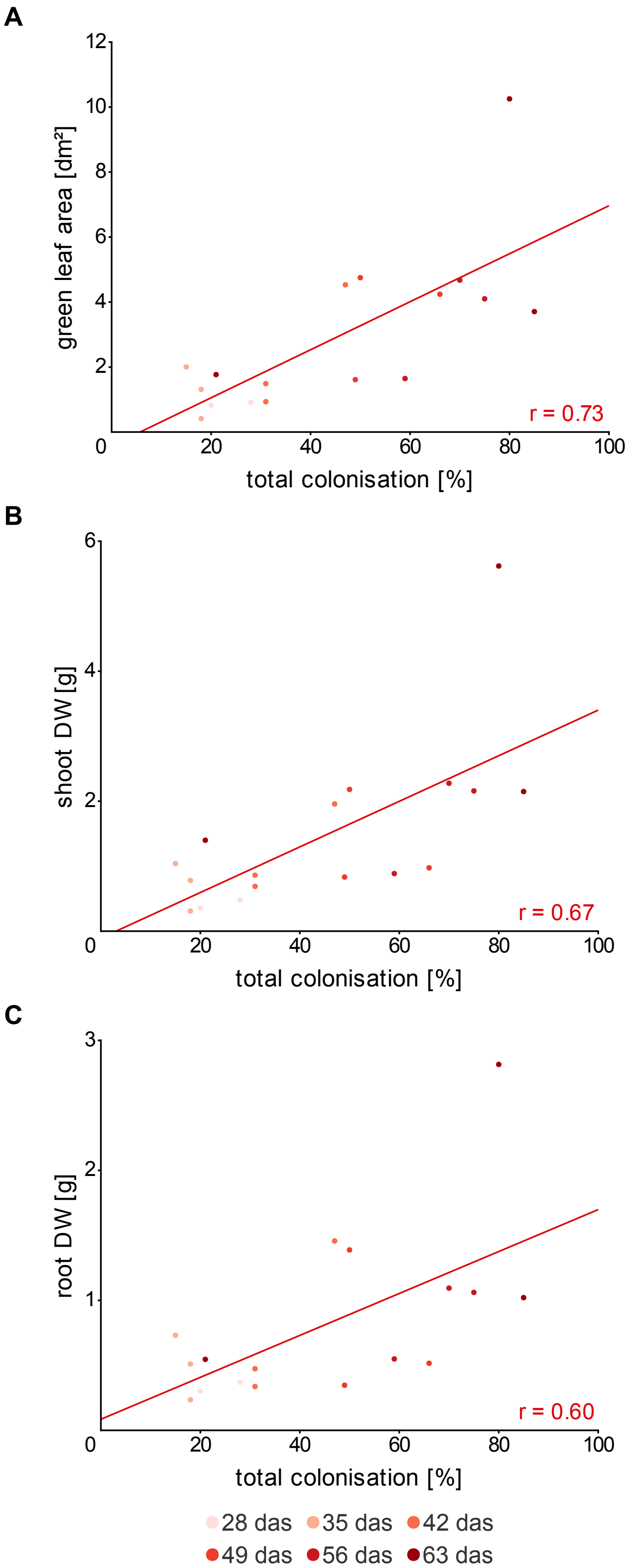


**Supplemental Figure S7.** Correlation of total root length colonization over time with plant growth parameters over time (experiment 3).

The percentage presence of any fungal structure (total colonization) was correlated with (A) green leaf area, (B) shoot dry weight and (C) root dry weight. Pearson correlation coefficients are indicated in red in the lower right corner of each plot, while full statistics are reported here: (A) $r = 0.73$, $p < 0.001$, $n = 17$, 95% confidence level [0.38, 0.90]; (B) $r = 0.67$, $p = 0.004$, $n = 17$, 95% confidence level [0.27, 0.87]; (C) $r = 0.60$, $p = 0.010$, $n = 17$, 95% confidence level [0.17, 0.84].

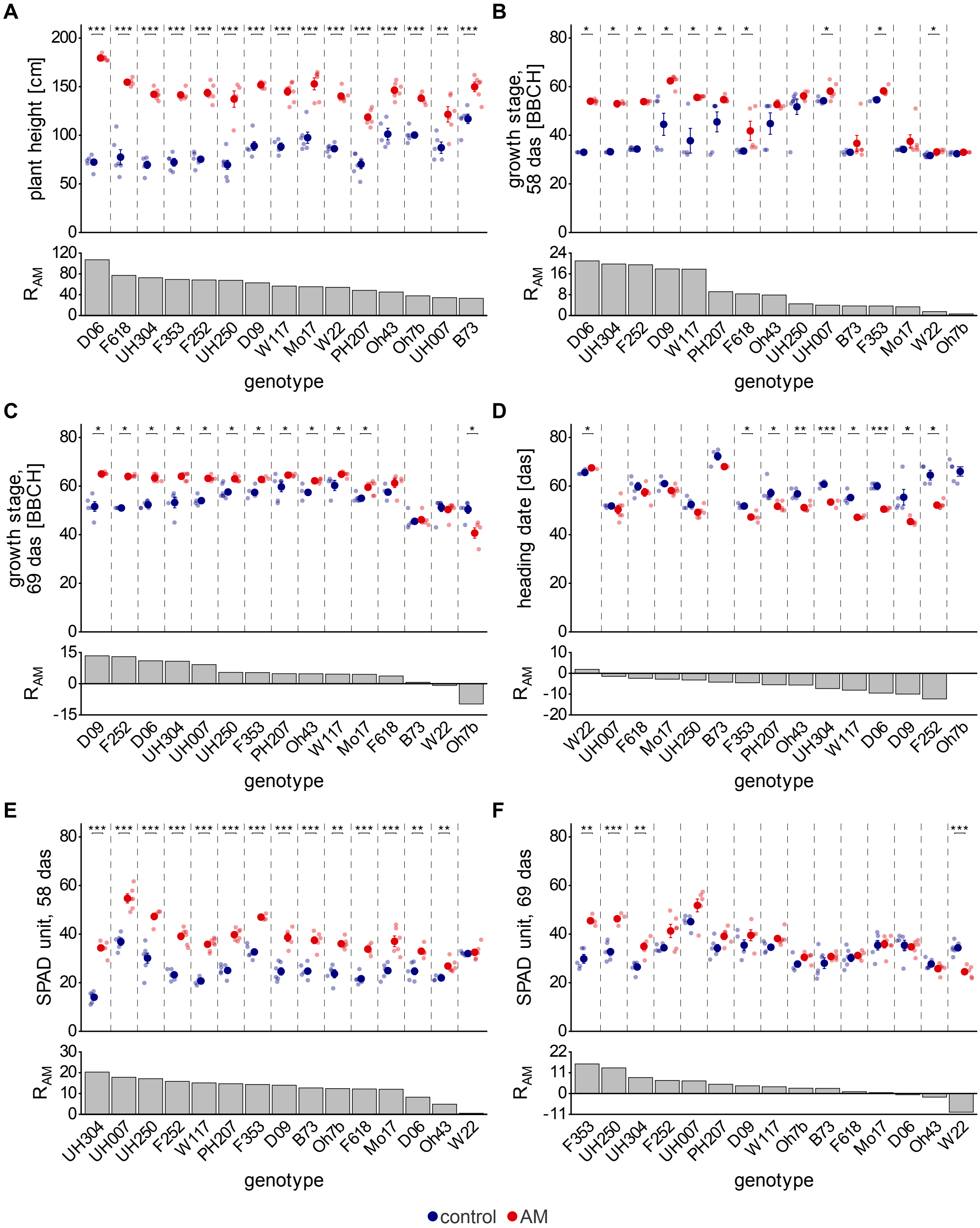


**Supplemental Figure S8.** Growth parameters and chlorophyll content across 15 maize genotypes grown with or without the AM fungus *R. irregularis* (experiment 1).

(A) Plant height, (B) growth stage on the BBCH scale at 58 days after sowing (das), (C) growth stage on the BBCH scale at 69 das, (D) heading date, (E) chlorophyll estimate by SPAD measurement at 58 das and (F) chlorophyll estimate at 69 das. The upper panels show values from individual plants (light-colored dots) along with the means (dark-colored dots) and standard errors of the mean for each genotype and treatment combination. Asterisks denote significant differences between treatment means within a genotype (n = 0-7, t-test (Mann–Whitney U test for growth stage measurements), *: $p < 0.05$; **: $p < 0.01$; ***: $p < 0.001$). The lower panel shows genotype-specific $R_{AM}$, calculated as the difference between the means of AM-treated plants and control plants. In all panels genotypes are displayed in decreasing order of $R_{AM}$.

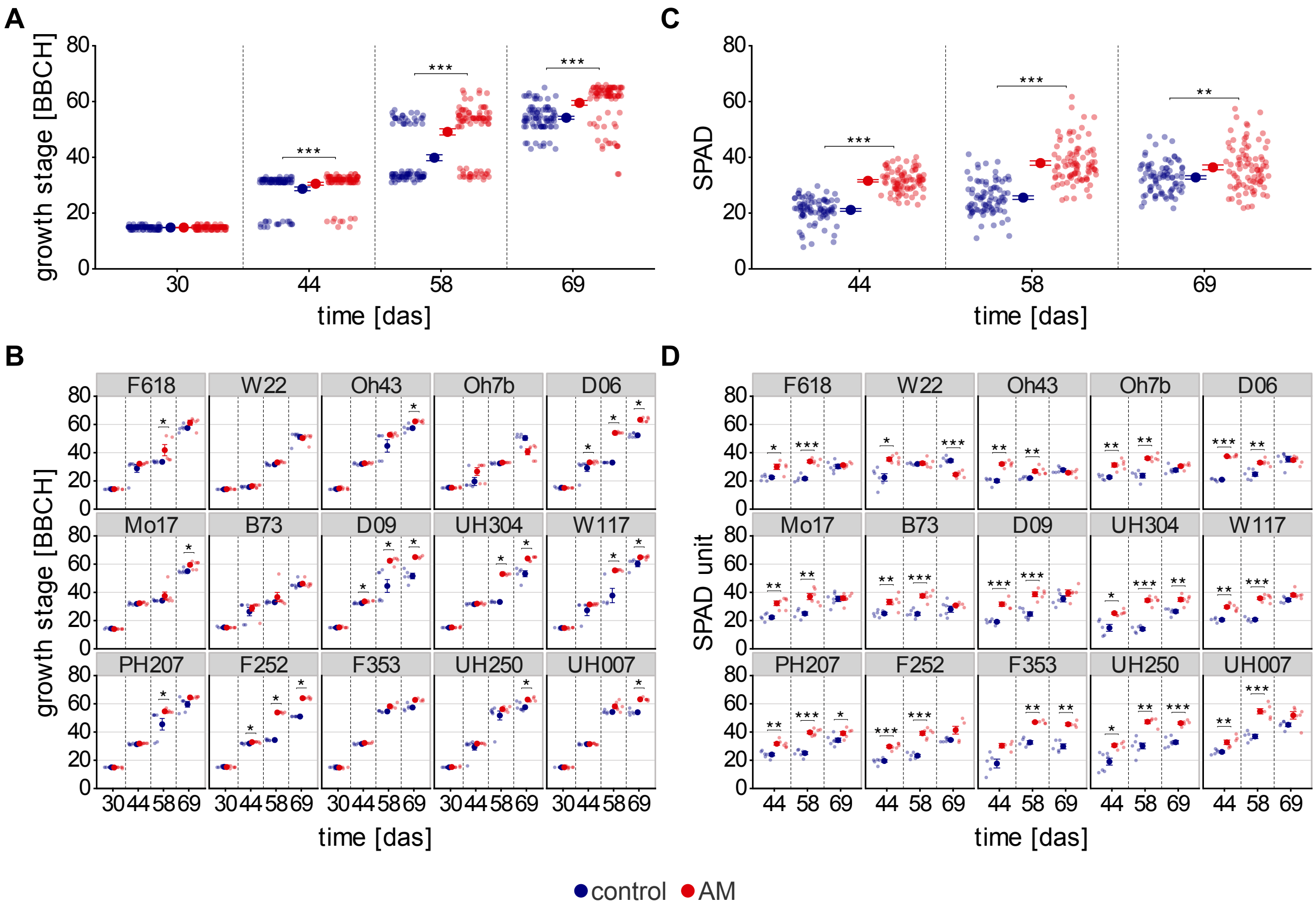


**Supplemental Figure S9.** $R_{AM}$ for growth stage and chlorophyll content estimates vary among maize lines across three timepoints (experiment 1).

(A) Growth stage on the BBCH scale for all 15 genotypes taken together and (B) for each individual genotype with and without AM. (C) Chlorophyll content estimate in SPAD units for all 15 genotypes taken together, and (D) for each individual genotype. Individual plant data points (light color) are shown along with the means and standard error of the mean (dark color) for each genotype and treatment combination. Asterisks denote significant differences between treatments (n = 4-7, t-test for SPAD measurements and Mann–Whitney U test for growth stage measurements), *: $p < 0.05$; **: $p < 0.01$; ***: $p < 0.001$).

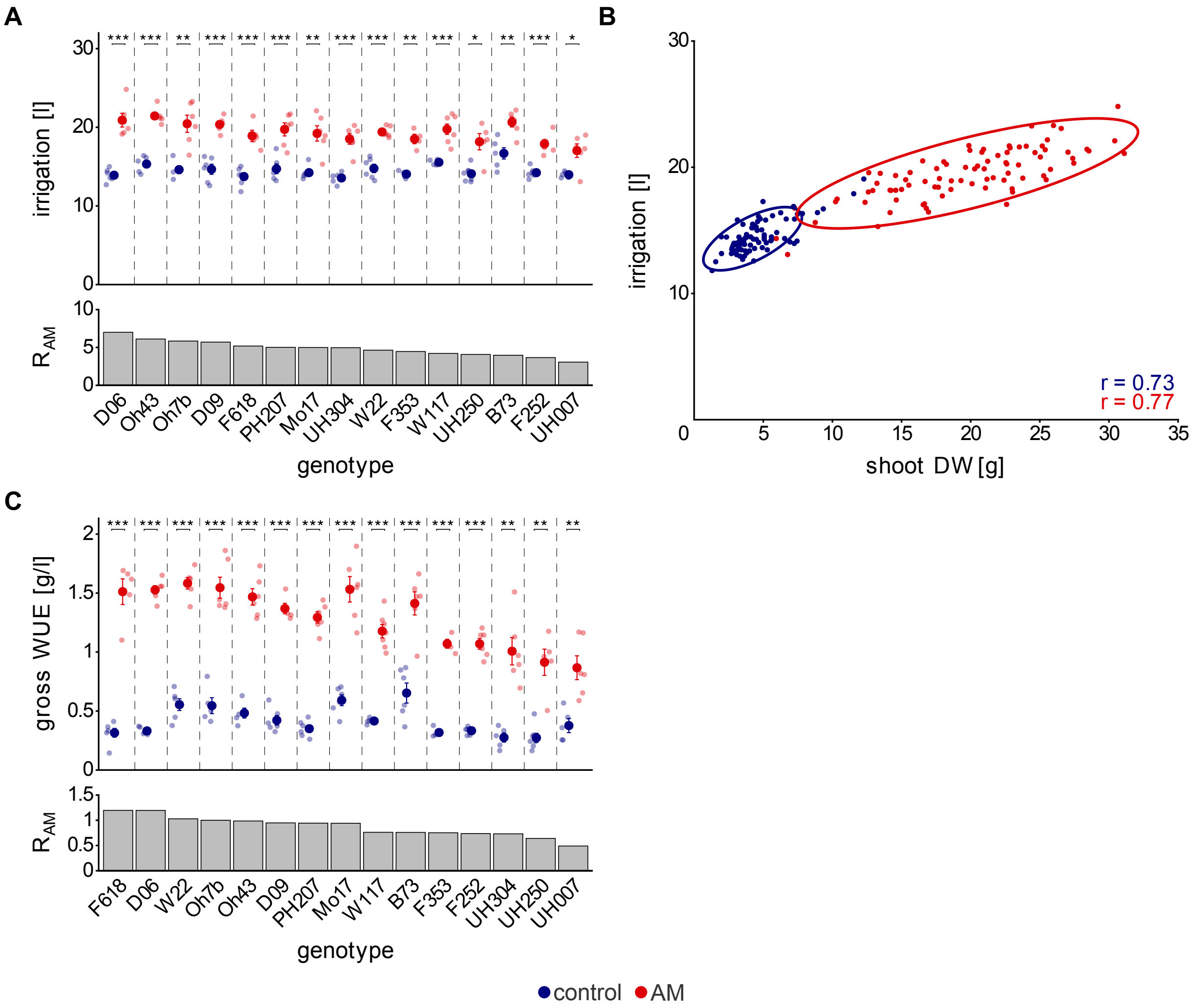


**Supplemental Figure S10.** Irrigation and gross water use efficiency (WUE) of 15 maize lines in experiment 1. (A) Total amount of water irrigated throughout the experiment, (B) correlation between irrigation amount and shoot dry weight and (C) gross WUE measured as the total dry matter produced per liter of irrigated water for each genotype x treatment combination. The upper panels show values from individual plants (light-colored dots) along with the means (dark-colored dots) and standard errors of the mean for each genotype and treatment combination. Asterisks denote significant differences between treatment averages within a genotype (n = 4-7, t-test, *: $p < 0.05$; **: $p < 0.01$; ***: $p < 0.001$). The lower panels display the AM response. Pearson correlation coefficients indicated in red for AM plants and blue for control plants are in the lower right corner of the correlation plot (B; control: r = 0.73, p = <0.001, n = 84, 95% confidence level [0.61, 0.82]; AM: r = 0.77, p = <0.001, n = 86, 95% confidence level [0.67, 0.85]).

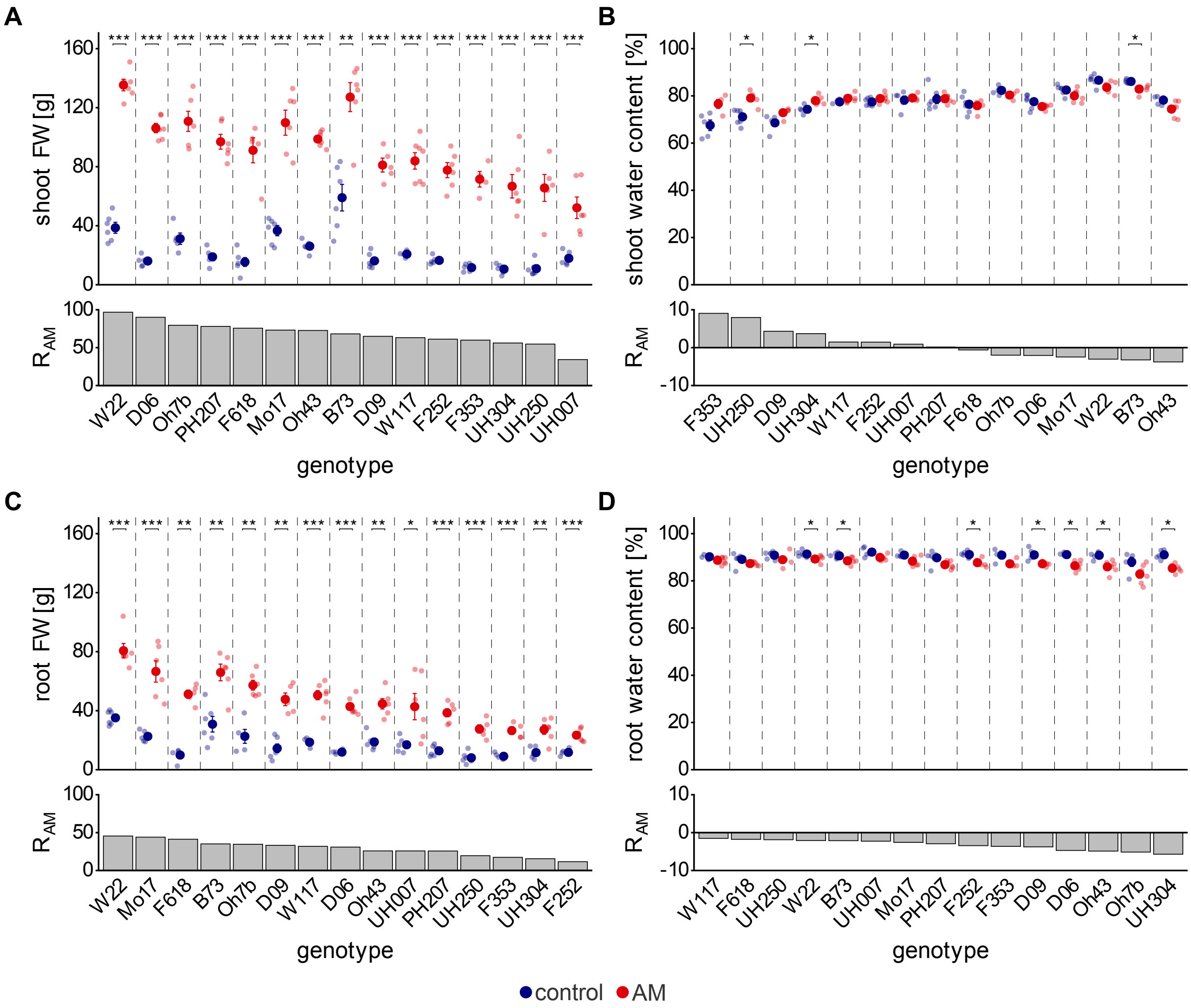


**Supplemental Figure S11.** Fresh weight and water content of root and shoot tissue in experiment 1.
(A) Shoot fresh weight, (B) shoot water content, (C) root fresh weight and (D) root water content. The upper panels show values from individual plants (light-colored dots) along with the means (dark-colored dots) and standard errors of the mean for each genotype. Asterisks denote significant differences between treatment means within a genotype and treatment combination (n = 4-7, t-test, *: $p < 0.05$; **: $p < 0.01$; ***: $p < 0.001$). The lower panel shows genotype-specific $R_{AM}$, calculated as the difference between the means of AM-treated plants and control plants. In all panels genotypes are displayed in decreasing order of $R_{AM}$.

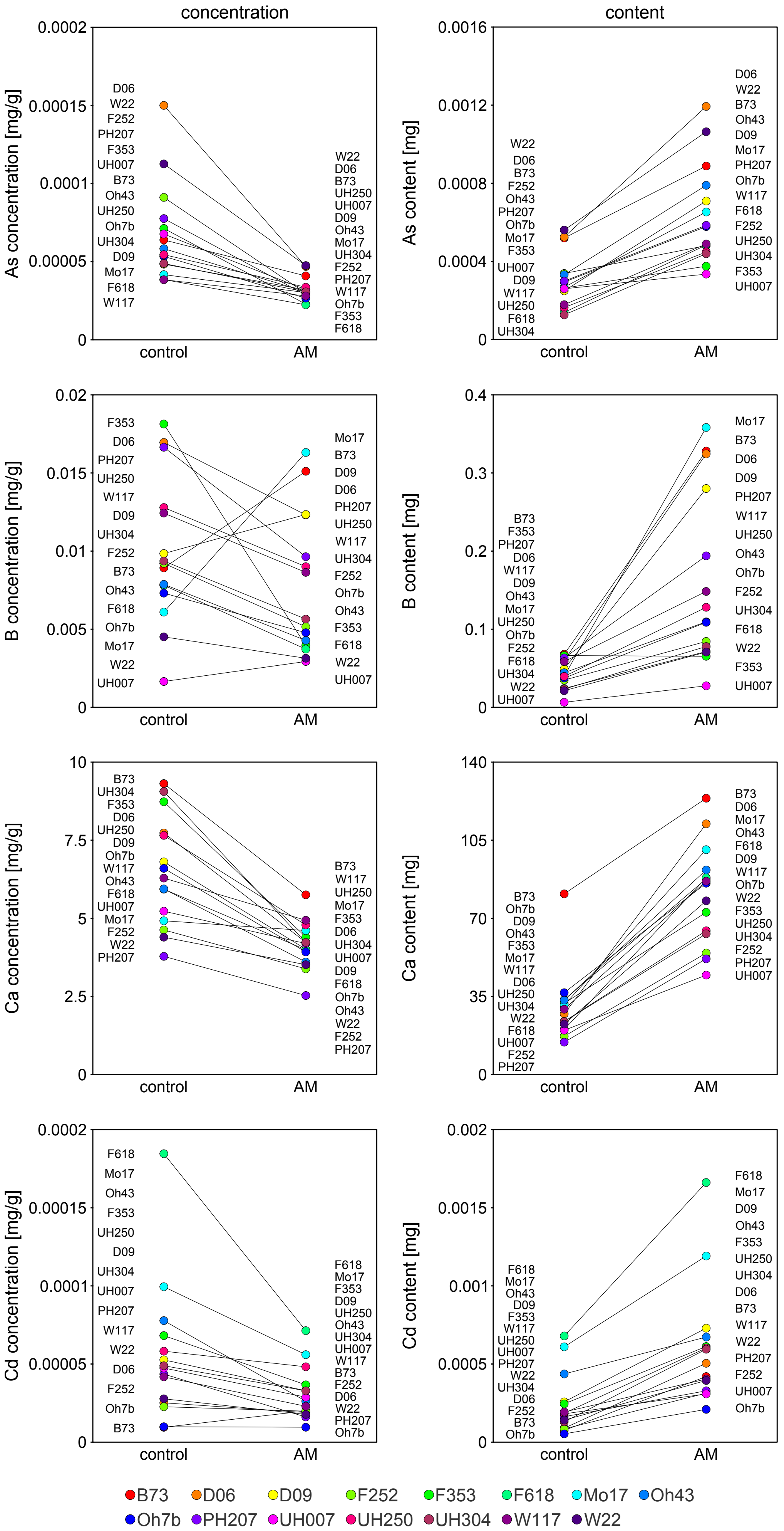

concentration
content
As concentration [mg/g]
0.0002
0.00015
0.0001
0.00005
0
D06
W22
F252
PH207
F353
UH007
B73
Oh43
UH250
Oh7b
UH304
D09
Mo17
F618
W117
W22
D06
B73
UH250
UH007
D09
Oh43
Mo17
UH304
F252
PH207
W117
Oh7b
F353
F618
control
AM
As content [mg]
0.0016
0.0012
0.0008
0.0004
0
W22
D06
B73
F252
Oh43
PH207
Oh7b
Mo17
F353
UH007
D09
W117
UH250
F618
UH304
D06
W22
B73
Oh43
D09
Mo17
PH207
Oh7b
W117
F618
F252
UH250
UH304
F353
UH007
control
AM
B concentration [mg/g]
0.02
0.015
0.01
0.005
0
F353
D06
PH207
UH250
W117
D09
UH304
F252
B73
Oh43
F618
Oh7b
Mo17
W22
UH007
Mo17
B73
D09
D06
PH207
UH250
W117
UH304
F252
Oh7b
Oh43
F353
F618
W22
UH007
control
AM
B content [mg]
0.4
0.3
0.2
0.1
0
B73
F353
PH207
D06
W117
D09
Oh43
Mo17
UH250
Oh7b
F252
F618
UH304
W22
UH007
Mo17
B73
D06
D09
PH207
W117
UH250
Oh43
Oh7b
F252
UH304
F618
W22
F353
UH007
control
AM
Ca concentration [mg/g]
10
7.5
5
2.5
0
B73
UH304
F353
D06
UH250
D09
Oh7b
W117
Oh43
F618
UH007
Mo17
F252
W22
PH207
B73
W117
UH250
Mo17
F353
D06
UH304
UH007
D09
F618
Oh7b
Oh43
W22
F252
PH207
control
AM
Ca content [mg]
140
105
70
35
0
B73
Oh7b
D09
Oh43
F353
Mo17
W117
D06
UH250
UH304
W22
F618
UH007
F252
PH207
B73
D06
Mo17
Oh43
F618
D09
W117
Oh7b
W22
F353
UH250
UH304
F252
PH207
UH007
control
AM
Cd concentration [mg/g]
0.0002
0.00015
0.0001
0.00005
0
F618
Mo17
Oh43
F353
UH250
D09
UH304
UH007
PH207
W117
W22
D06
F252
Oh7b
B73
F618
Mo17
F353
D09
UH250
Oh43
UH304
UH007
W117
B73
F252
D06
W22
PH207
Oh7b
control
AM
Cd content [mg]
0.002
0.0015
0.001
0.0005
0
F618
Mo17
Oh43
D09
F353
W117
UH250
UH007
PH207
W22
UH304
D06
F252
B73
Oh7b
F618
Mo17
D09
Oh43
F353
UH250
UH304
D06
B73
W117
W22
PH207
F252
UH007
Oh7b
control
AM
B73
D06
D09
F252
F353
F618
Mo17
Oh43
Oh7b
PH207
UH007
UH250
UH304
W117
W22

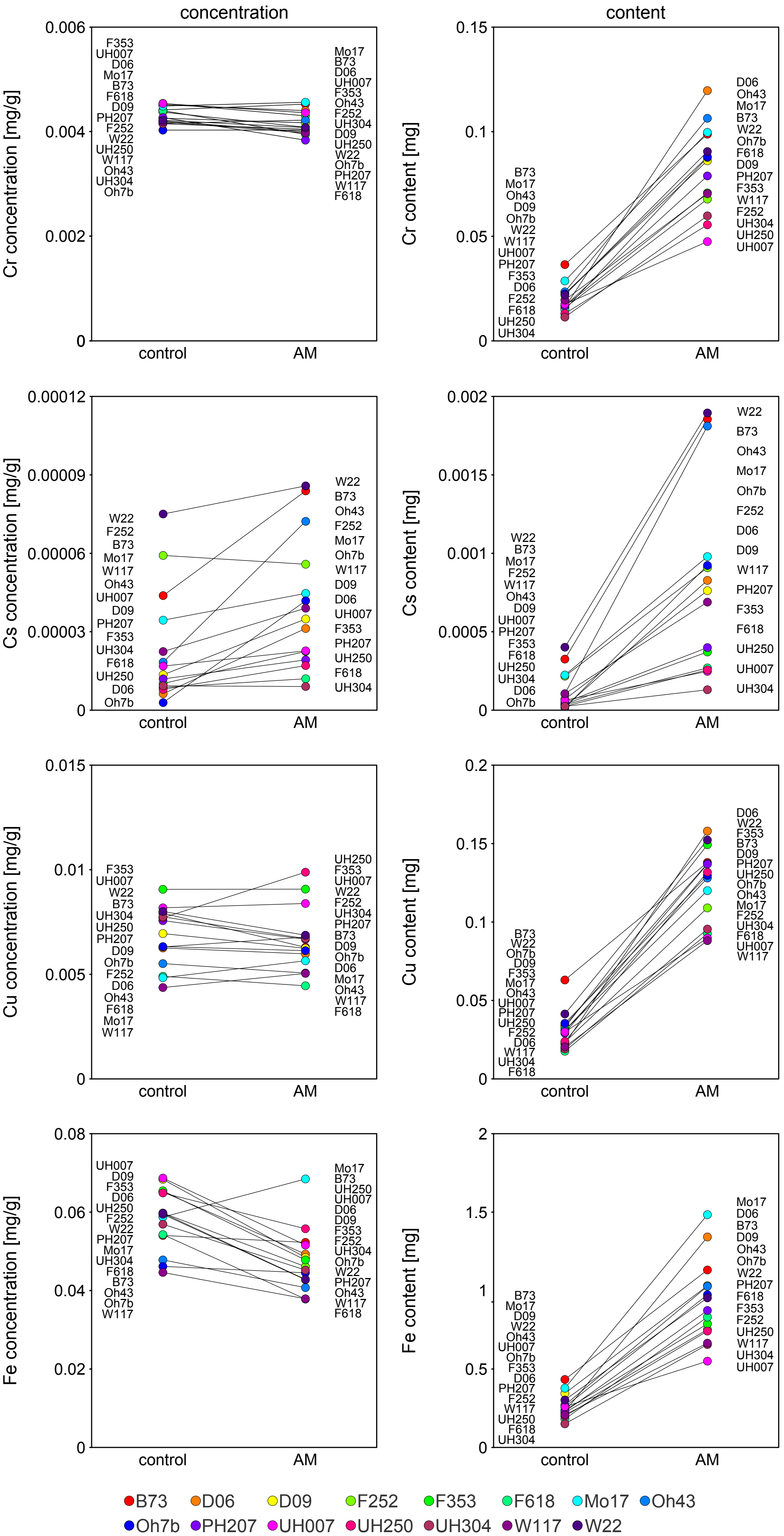

concentration
content
Cr concentration [mg/g]
0.006
0.004
0.002
0
F353 UH007 D06 Mo17 B73 F618 D09 PH207 F252 W22 UH250 W117 Oh43 UH304 Oh7b
Mo17 B73 D06 UH007 F353 Oh43 F252 UH304 D09 UH250 W22 Oh7b PH207 W117 F618
control
AM
Cr content [mg]
0.15
0.1
0.05
0
B73 Mo17 Oh43 D09 Oh7b W22 W117 UH007 PH207 F353 D06 F252 F618 UH250 UH304
D06 Oh43 Mo17 B73 W22 Oh7b F618 D09 PH207 F353 W117 F252 UH304 UH250 UH007
Cs concentration [mg/g]
0.00012
0.00009
0.00006
0.00003
0
W22 F252 B73 Mo17 W117 Oh43 UH007 D09 PH207 F353 UH304 F618 UH250 D06 Oh7b
W22 B73 Oh43 F252 Mo17 Oh7b W117 D09 D06 UH007 F353 PH207 UH250 F618 UH304
Cs content [mg]
0.002
0.0015
0.001
0.0005
0
W22 B73 Mo17 F252 W117 Oh43 D09 UH007 PH207 F353 F618 UH250 UH304 D06 Oh7b
W22 B73 Oh43 Mo17 Oh7b F252 D06 D09 W117 PH207 F353 F618 UH250 UH007 UH304
Cu concentration [mg/g]
0.015
0.01
0.005
0
F353 UH007 W22 B73 UH304 UH250 PH207 D09 Oh7b F252 D06 Oh43 F618 Mo17 W117
UH250 F353 UH007 W22 F252 UH304 PH207 B73 D09 Oh7b D06 Mo17 Oh43 W117 F618
Cu content [mg]
0.2
0.15
0.1
0.05
0
B73 W22 Oh7b D09 F353 Mo17 Oh43 UH007 PH207 UH250 F252 D06 W117 UH304 F618
D06 W22 F353 B73 D09 PH207 UH250 Oh7b Oh43 Mo17 F252 UH304 F618 UH007 W117
Fe concentration [mg/g]
0.08
0.06
0.04
0.02
0
UH007 D09 F353 D06 UH250 F252 W22 PH207 Mo17 UH304 F618 B73 Oh43 Oh7b W117
Mo17 B73 UH250 UH007 D06 D09 F353 F252 UH304 Oh7b W22 PH207 Oh43 W117 F618
Fe content [mg]
2
1.5
1
0.5
0
B73 Mo17 D09 W22 Oh43 UH007 Oh7b F353 D06 PH207 F252 W117 UH250 F618 UH304
Mo17 D06 B73 D09 Oh43 Oh7b W22 PH207 F618 F353 F252 UH250 W117 UH304 UH007
B73 D06 D09 F252 F353 F618 Mo17 Oh43
Oh7b PH207 UH007 UH250 UH304 W117 W22

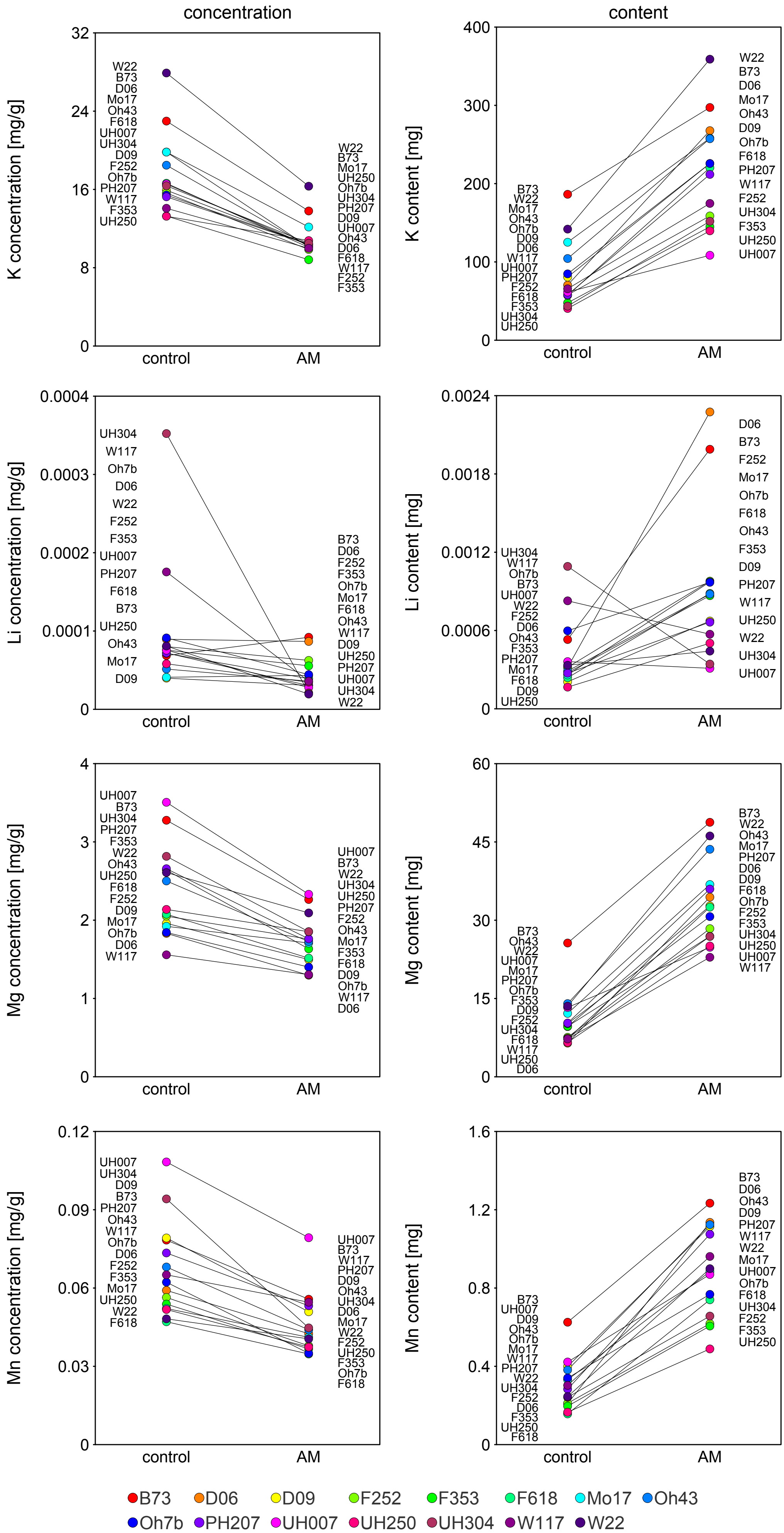
concentration
content
K concentration [mg/g]
K content [mg]
Li concentration [mg/g]
Li content [mg]
Mg concentration [mg/g]
Mg content [mg]
Mn concentration [mg/g]
Mn content [mg]
control
AM
B73 D06 D09 F252 F353 F618 Mo17 Oh43
Oh7b PH207 UH007 UH250 UH304 W117 W22

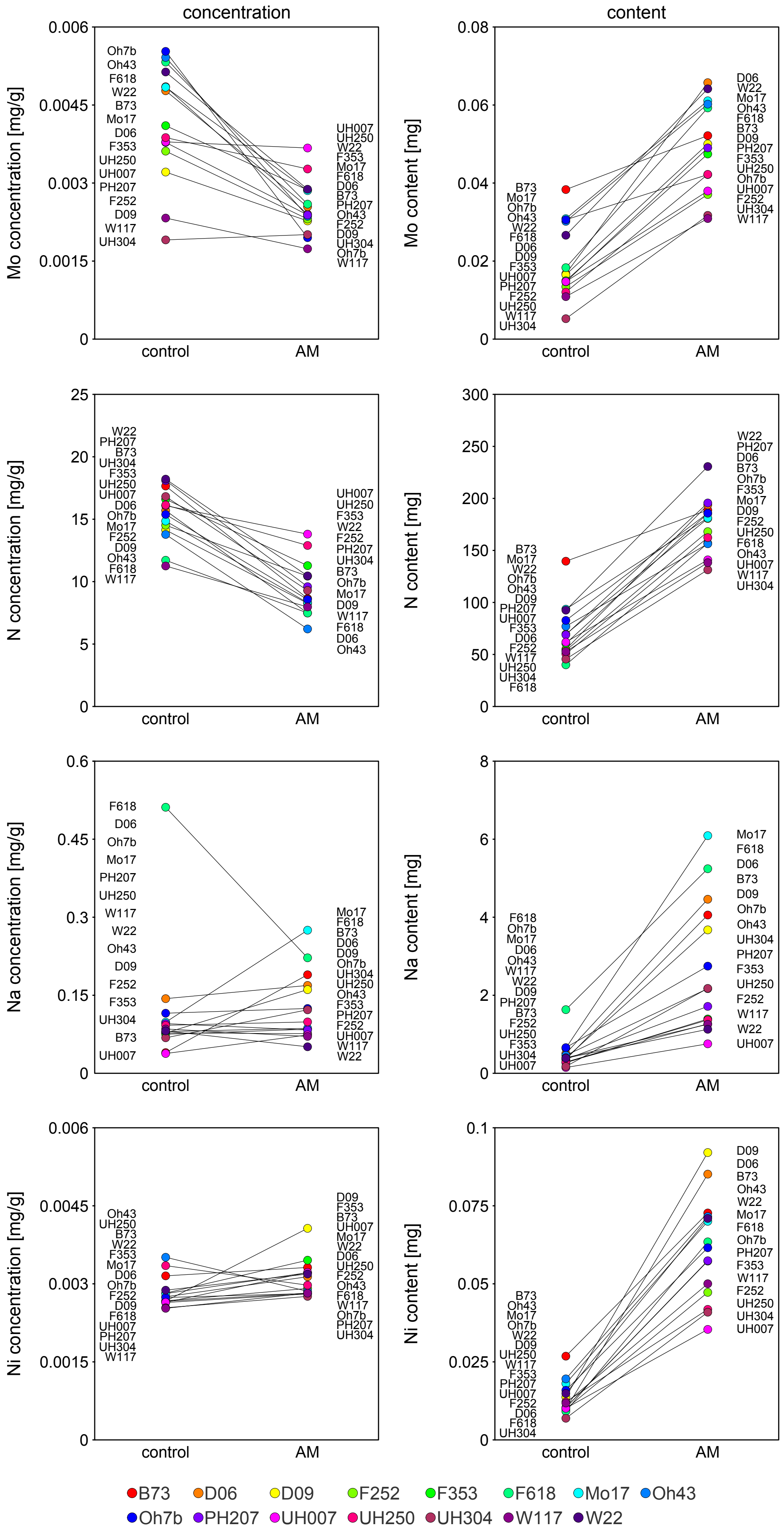
concentration
content
Mo concentration [mg/g]
0
0.0015
0.003
0.0045
0.006
Mo content [mg]
0
0.02
0.04
0.06
0.08
N concentration [mg/g]
0
5
10
15
20
25
N content [mg]
0
50
100
150
200
250
300
Na concentration [mg/g]
0
0.15
0.3
0.45
0.6
Na content [mg]
0
2
4
6
8
Ni concentration [mg/g]
0
0.0015
0.003
0.0045
0.006
Ni content [mg]
0
0.025
0.05
0.075
0.1
control
AM
B73
D06
D09
F252
F353
F618
Mo17
Oh43
Oh7b
PH207
UH007
UH250
UH304
W117
W22

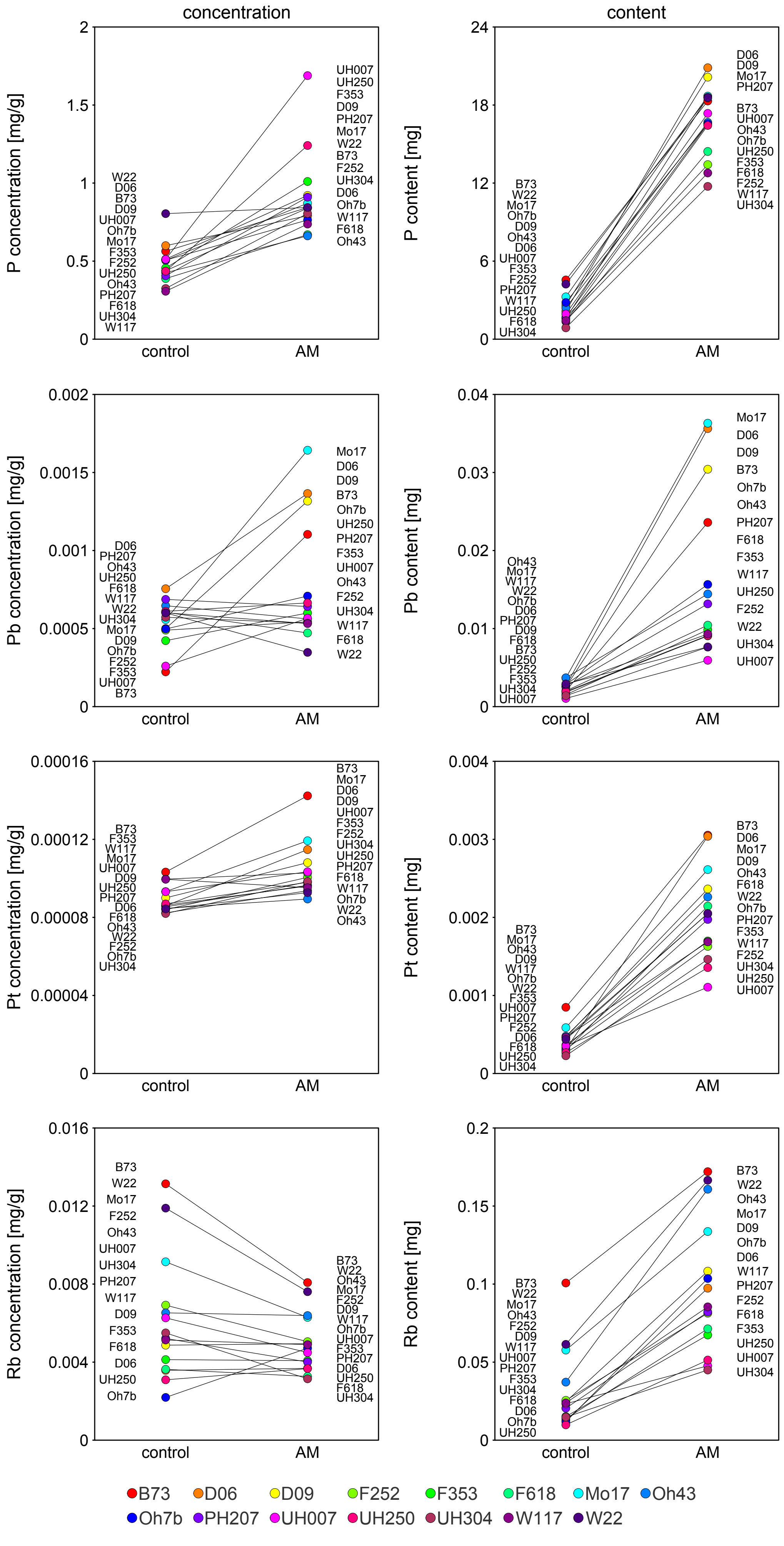

concentration
content
P concentration [mg/g]
P content [mg]
Pb concentration [mg/g]
Pb content [mg]
Pt concentration [mg/g]
Pt content [mg]
Rb concentration [mg/g]
Rb content [mg]
control
AM
B73 D06 D09 F252 F353 F618 Mo17 Oh43
Oh7b PH207 UH007 UH250 UH304 W117 W22

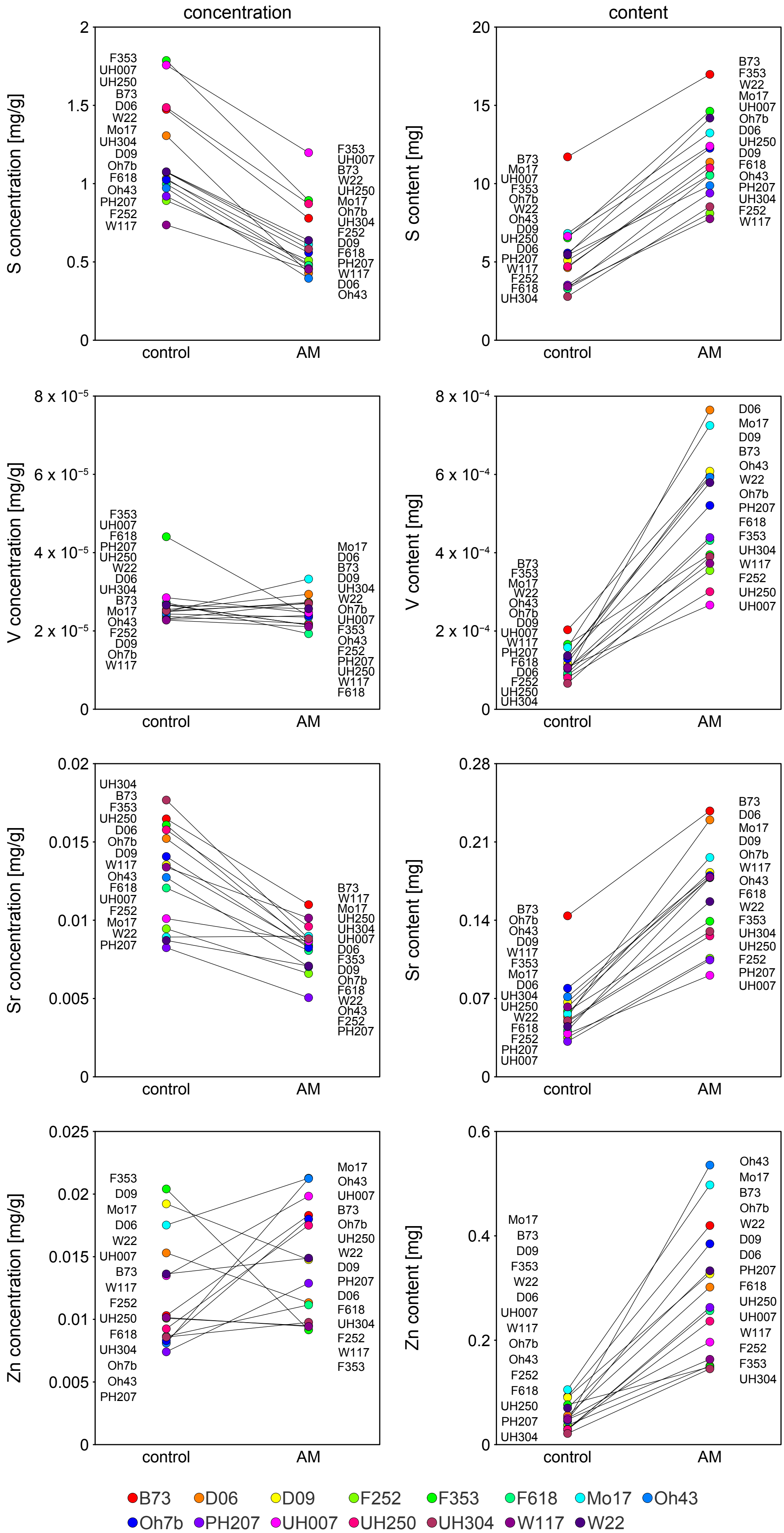
concentration
content
S concentration [mg/g]
S content [mg]
V concentration [mg/g]
V content [mg]
Sr concentration [mg/g]
Sr content [mg]
Zn concentration [mg/g]
Zn content [mg]
control
AM
0
0.5
1
1.5
2
5
10
15
20
2 x 10⁻⁵
4 x 10⁻⁵
6 x 10⁻⁵
8 x 10⁻⁵
2 x 10⁻⁴
4 x 10⁻⁴
6 x 10⁻⁴
8 x 10⁻⁴
0.005
0.01
0.015
0.02
0.07
0.14
0.21
0.28
0.025
0.2
0.4
0.6
B73 D06 D09 F252 F353 F618 Mo17 Oh43
Oh7b PH207 UH007 UH250 UH304 W117 W22

**Supplemental Figure S12.** Impact of AM on uptake of 24 elements by 15 maize inbred lines in experiment 1.
Mean values of shoot concentration [mg/g] (left) and total shoot content [mg] (right) of element ions are shown for each genotype by color coded circles. Lines connecting the datapoints between control and AM treatment indicate the change.

**Supplemental Table S1.** Timepoint of the first significant differences in AM responsiveness ($R_{AM}$) of shoot area (experiment 1).
The first timepoint in days after sowing (das) at which inoculated (AM) and control plants differed significantly for each genotype are shown. Significance was determined using a linear mixed-effects model based on estimated marginal means, with plant identity included as a random effect to account for repeated measurements. P-values were adjusted for multiple testing using the false discovery rate (FDR), and the first time point with an adjusted p-value < 0.05 is reported here.

| genotype | first significance between AM and control [das] |
|---|---|
| F618 | 41 |
| W22 | 40 |
| Oh43 | 40 |
| Oh7b | 41 |
| D06 | 40 |
| Mo17 | 40 |
| B73 | 50 |
| D09 | 40 |
| UH304 | 50 |
| W117 | 42 |
| PH207 | 41 |
| F252 | 41 |
| F353 | 40 |
| UH250 | 41 |
| UH007 | 50 |

**Supplemental Table S2.** Timepoint of the first significant differences in AM responsiveness ($R_{AM}$) of shoot area (experiment 2).
The first timepoint in days after sowing (das) at which inoculated (AM) and control plants differed significantly for each genotype are shown. Significance was determined using a linear mixed-effects model based on estimated marginal means, with plant identity included as a random effect to account for repeated measurements. P-values were adjusted for multiple testing using the false discovery rate (FDR), and the first time point with an adjusted p-value < 0.05 is reported here.

| genotype | first significance between AM and control [das] |
|---|---|
| D06 | 40 |
| F353 | 42 |

**Supplemental Table S3.** R-values of correlations between colonisation parameters and $R_{AM}$ for three growth traits (experiment 3).

| | total colonisation | arbuscules | vesicles |
|---|---|---|---|
| $R_{AM}$ root DW | 0.68 | 0.75 | 0.69 |
| $R_{AM}$ shoot DW | 0.65 | 0.76 | 0.69 |
| $R_{AM}$ green leaf area | 0.70 | 0.79 | 0.74 |

**Supplemental Table S4.** R-values of correlations between $R_{AM}$ of chlorophyll content and $R_{AM}$ of shoot and root dry weight (experiment 1).

| | **$R_{AM}$ shoot DW** | **$R_{AM}$ root DW** |
|---|---|---|
| $R_{AM}$ SPAD unit, 58 das | -0.68 | -0.56 |
| $R_{AM}$ SPAD unit, 69 das | -0.62 | -0.68 |

**Supplemental Table S5.** Cross-trait predictability of RAM across genotypes. Ridge regression models were used to predict the RAM of each response trait from RAM values of all remaining traits, only growth-related traits, or only ionome-related traits. Predictive performance is shown as leave-one-out cross-validated $R^2$. P-perm denotes the empirical permutation-test P-value and adj-p-value the Benjamini-Hochberg adjusted P-value. Bootstrap_CI_lower_95 and bootstrap_CI_upper_95 indicate exploratory 95% bootstrap intervals for $R^2$_LOOCV. Negative $R^2$_LOOCV values indicate predictive performance worse than mean prediction.

| response trait | predictor set | N (genotypes) | N (predictors) | $R^2$_LOOCV | bootstrap_CI_lower_95 | bootstrap_CI_upper_95 | P-perm | adj-p-value |
|---|---|---|---|---|---|---|---|---|
| $R_{AM}$ As concentration | All | 15 | 32 | -0.150 | -0.149 | 0.362 | 0.745 | 0.768 |
| $R_{AM}$ As concentration | Growth | 15 | 6 | -0.149 | -0.151 | 0.754 | 0.582 | 0.647 |
| $R_{AM}$ As concentration | Ionome | 15 | 26 | -0.149 | -0.149 | 0.308 | 0.656 | 0.714 |
| $R_{AM}$ B concentration | All | 15 | 32 | 0.116 | -0.135 | 0.486 | 0.006 | 0.031 |
| $R_{AM}$ B concentration | Growth | 15 | 6 | 0.159 | -0.105 | 0.756 | 0.059 | 0.139 |
| $R_{AM}$ B concentration | Ionome | 15 | 26 | 0.099 | -0.146 | 0.473 | 0.014 | 0.057 |
| $R_{AM}$ Ca concentration | All | 15 | 32 | -0.002 | -0.074 | 0.409 | 0.121 | 0.222 |
| $R_{AM}$ Ca concentration | Growth | 15 | 6 | -0.095 | -0.150 | 0.733 | 0.263 | 0.368 |
| $R_{AM}$ Ca concentration | Ionome | 15 | 26 | -0.009 | -0.072 | 0.394 | 0.108 | 0.214 |
| $R_{AM}$ Cd concentration | All | 15 | 32 | -0.150 | -0.153 | 0.312 | 0.898 | 0.906 |
| $R_{AM}$ Cd concentration | Growth | 15 | 6 | -0.149 | -0.151 | 0.774 | 0.678 | 0.720 |
| $R_{AM}$ Cd concentration | Ionome | 15 | 26 | -0.150 | -0.153 | 0.271 | 0.906 | 0.906 |
| $R_{AM}$ Cr concentration | All | 15 | 32 | 0.071 | -0.112 | 0.451 | 0.045 | 0.117 |
| $R_{AM}$ Cr concentration | Growth | 15 | 6 | -0.147 | -0.151 | 0.682 | 0.457 | 0.565 |
| $R_{AM}$ Cr concentration | Ionome | 15 | 26 | 0.090 | -0.075 | 0.429 | 0.023 | 0.073 |
| $R_{AM}$ Cs concentration | All | 15 | 32 | 0.009 | -0.148 | 0.465 | 0.102 | 0.206 |
| $R_{AM}$ Cs concentration | Growth | 15 | 6 | 0.008 | -0.149 | 0.829 | 0.170 | 0.276 |
| $R_{AM}$ Cs concentration | Ionome | 15 | 26 | -0.042 | -0.148 | 0.377 | 0.135 | 0.242 |
| $R_{AM}$ Cu concentration | All | 15 | 32 | 0.065 | -0.077 | 0.469 | 0.027 | 0.081 |
| $R_{AM}$ Cu concentration | Growth | 15 | 6 | -0.089 | -0.150 | 0.640 | 0.264 | 0.368 |
| $R_{AM}$ Cu concentration | Ionome | 15 | 26 | 0.032 | -0.078 | 0.400 | 0.049 | 0.124 |
| $R_{AM}$ Fe concentration | All | 15 | 32 | 0.025 | -0.115 | 0.439 | 0.063 | 0.145 |
| $R_{AM}$ Fe concentration | Growth | 15 | 6 | -0.085 | -0.149 | 0.705 | 0.252 | 0.368 |
| $R_{AM}$ Fe concentration | Ionome | 15 | 26 | 0.007 | -0.101 | 0.419 | 0.081 | 0.178 |
| $R_{AM}$ growth stage, 69 das | All | 15 | 32 | -0.074 | -0.122 | 0.363 | 0.200 | 0.309 |
| $R_{AM}$ growth stage, 69 das | Growth | 15 | 5 | 0.256 | -0.124 | 0.890 | 0.021 | 0.069 |
| $R_{AM}$ growth stage, 69 das | Ionome | 15 | 27 | -0.132 | -0.147 | 0.305 | 0.358 | 0.486 |
| $R_{AM}$ plant height | All | 15 | 32 | -0.107 | -0.139 | 0.434 | 0.259 | 0.368 |
| $R_{AM}$ plant height | Growth | 15 | 5 | 0.157 | -0.149 | 0.844 | 0.035 | 0.099 |
| $R_{AM}$ plant height | Ionome | 15 | 27 | -0.148 | -0.148 | 0.391 | 0.464 | 0.565 |
| $R_{AM}$ K concentration | All | 15 | 32 | 0.161 | -0.008 | 0.491 | 0.007 | 0.035 |
| $R_{AM}$ K concentration | Growth | 15 | 6 | 0.223 | -0.088 | 0.850 | 0.051 | 0.126 |
| $R_{AM}$ K concentration | Ionome | 15 | 26 | 0.120 | -0.062 | 0.419 | 0.010 | 0.043 |
| $R_{AM}$ Li concentration | All | 15 | 32 | -0.148 | -0.149 | 0.365 | 0.560 | 0.637 |
| $R_{AM}$ Li concentration | Growth | 15 | 6 | -0.148 | -0.150 | 0.597 | 0.578 | 0.647 |
| $R_{AM}$ Li concentration | Ionome | 15 | 26 | -0.148 | -0.148 | 0.348 | 0.528 | 0.622 |
| $R_{AM}$ Mg concentration | All | 15 | 32 | -0.010 | -0.115 | 0.429 | 0.142 | 0.247 |
| $R_{AM}$ Mg concentration | Growth | 15 | 6 | -0.149 | -0.151 | 0.655 | 0.557 | 0.637 |
| $R_{AM}$ Mg concentration | Ionome | 15 | 26 | -0.013 | -0.077 | 0.401 | 0.111 | 0.215 |
| $R_{AM}$ Mn concentration | All | 15 | 32 | -0.147 | -0.149 | 0.358 | 0.424 | 0.538 |
| $R_{AM}$ Mn concentration | Growth | 15 | 6 | -0.148 | -0.150 | 0.692 | 0.483 | 0.576 |
| $R_{AM}$ Mn concentration | Ionome | 15 | 26 | -0.143 | -0.148 | 0.320 | 0.376 | 0.500 |
| $R_{AM}$ Mo concentration | All | 15 | 32 | 0.277 | 0.150 | 0.523 | 0.001 | 0.011 |
| $R_{AM}$ Mo concentration | Growth | 15 | 6 | 0.645 | 0.401 | 0.921 | 0.000 | 0.000 |
| $R_{AM}$ Mo concentration | Ionome | 15 | 26 | 0.100 | -0.004 | 0.406 | 0.028 | 0.082 |
| $R_{AM}$ N concentration | All | 15 | 32 | 0.217 | 0.008 | 0.536 | 0.004 | 0.025 |
| $R_{AM}$ N concentration | Growth | 15 | 6 | 0.018 | -0.149 | 0.786 | 0.154 | 0.254 |
| $R_{AM}$ N concentration | Ionome | 15 | 26 | 0.167 | -0.010 | 0.466 | 0.008 | 0.036 |
| $R_{AM}$ Na concentration | All | 15 | 32 | 0.034 | -0.126 | 0.420 | 0.016 | 0.059 |
| $R_{AM}$ Na concentration | Growth | 15 | 6 | -0.148 | -0.150 | 0.738 | 0.538 | 0.627 |
| $R_{AM}$ Na concentration | Ionome | 15 | 26 | 0.032 | -0.122 | 0.422 | 0.015 | 0.057 |
| $R_{AM}$ Ni concentration | All | 15 | 32 | -0.131 | -0.149 | 0.316 | 0.313 | 0.430 |
| $R_{AM}$ Ni concentration | Growth | 15 | 6 | -0.149 | -0.151 | 0.813 | 0.606 | 0.667 |
| $R_{AM}$ Ni concentration | Ionome | 15 | 26 | -0.107 | -0.148 | 0.276 | 0.255 | 0.368 |
| $R_{AM}$ $N0_3$ concentration | All | 15 | 32 | -0.007 | -0.119 | 0.438 | 0.121 | 0.222 |
| $R_{AM}$ $N0_3$ concentration | Growth | 15 | 6 | -0.148 | -0.151 | 0.596 | 0.434 | 0.544 |
| $R_{AM}$ $N0_3$ concentration | Ionome | 15 | 26 | -0.003 | -0.110 | 0.407 | 0.091 | 0.192 |
| $R_{AM}$ P concentration | All | 15 | 32 | 0.218 | 0.072 | 0.507 | 0.000 | 0.000 |
| $R_{AM}$ P concentration | Growth | 15 | 6 | 0.421 | 0.214 | 0.897 | 0.003 | 0.021 |
| $R_{AM}$ P concentration | Ionome | 15 | 26 | 0.081 | -0.022 | 0.364 | 0.004 | 0.025 |
| $R_{AM}$ Pb concentration | All | 15 | 32 | 0.026 | -0.109 | 0.429 | 0.067 | 0.151 |
| $R_{AM}$ Pb concentration | Growth | 15 | 6 | -0.149 | -0.151 | 0.745 | 0.682 | 0.720 |
| $R_{AM}$ Pb concentration | Ionome | 15 | 26 | 0.052 | -0.107 | 0.422 | 0.036 | 0.099 |
| $R_{AM}$ $P0_4$ concentration | All | 15 | 32 | 0.201 | 0.073 | 0.499 | 0.001 | 0.011 |
| $R_{AM}$ $P0_4$ concentration | Growth | 15 | 6 | 0.466 | 0.170 | 0.910 | 0.003 | 0.021 |
| $R_{AM}$ $P0_4$ concentration | Ionome | 15 | 26 | 0.064 | -0.015 | 0.401 | 0.008 | 0.036 |
| $R_{AM}$ Pt concentration | All | 15 | 32 | -0.015 | -0.147 | 0.492 | 0.114 | 0.217 |
| $R_{AM}$ Pt concentration | Growth | 15 | 6 | -0.150 | -0.151 | 0.631 | 0.842 | 0.859 |
| $R_{AM}$ Pt concentration | Ionome | 15 | 26 | 0.014 | -0.148 | 0.479 | 0.053 | 0.128 |
| $R_{AM}$ Rb concentration | All | 15 | 32 | -0.148 | -0.149 | 0.404 | 0.468 | 0.565 |
| $R_{AM}$ Rb concentration | Growth | 15 | 6 | -0.150 | -0.150 | 0.757 | 0.719 | 0.749 |
| $R_{AM}$ Rb concentration | Ionome | 15 | 26 | -0.134 | -0.148 | 0.372 | 0.379 | 0.500 |
| $R_{AM}$ root DW | All | 15 | 32 | 0.231 | 0.123 | 0.524 | 0.002 | 0.020 |
| $R_{AM}$ root DW | Growth | 15 | 5 | 0.552 | 0.292 | 0.923 | 0.003 | 0.021 |
| $R_{AM}$ root DW | Ionome | 15 | 27 | 0.086 | 0.004 | 0.459 | 0.015 | 0.057 |
| $R_{AM}$ S concentration | All | 15 | 32 | 0.013 | -0.147 | 0.421 | 0.089 | 0.192 |
| $R_{AM}$ S concentration | Growth | 15 | 6 | -0.149 | -0.150 | 0.644 | 0.684 | 0.720 |
| $R_{AM}$ S concentration | Ionome | 15 | 26 | 0.042 | -0.119 | 0.379 | 0.040 | 0.107 |
| $R_{AM}$ shoot DW | All | 15 | 32 | 0.200 | 0.017 | 0.509 | 0.005 | 0.029 |
| $R_{AM}$ shoot DW | Growth | 15 | 5 | 0.534 | 0.196 | 0.922 | 0.003 | 0.021 |
| $R_{AM}$ shoot DW | Ionome | 15 | 27 | 0.084 | -0.054 | 0.413 | 0.025 | 0.077 |
| $R_{AM}$ shoot area | All | 15 | 32 | 0.367 | 0.210 | 0.565 | 0.000 | 0.000 |
| $R_{AM}$ shoot area | Growth | 15 | 5 | 0.781 | 0.665 | 0.956 | 0.000 | 0.000 |
| $R_{AM}$ shoot area | Ionome | 15 | 27 | 0.199 | 0.066 | 0.463 | 0.001 | 0.011 |
| $R_{AM}$ $S0_4$ concentration | All | 15 | 32 | -0.048 | -0.148 | 0.436 | 0.137 | 0.242 |
| $R_{AM}$ $S0_4$ concentration | Growth | 15 | 6 | -0.145 | -0.151 | 0.692 | 0.407 | 0.528 |
| $R_{AM}$ $S0_4$ concentration | Ionome | 15 | 26 | -0.043 | -0.139 | 0.366 | 0.097 | 0.200 |
| $R_{AM}$ SPAD unit, 69 das | All | 15 | 32 | 0.273 | 0.088 | 0.544 | 0.000 | 0.000 |
| $R_{AM}$ SPAD unit, 69 das | Growth | 15 | 5 | 0.392 | 0.205 | 0.884 | 0.006 | 0.031 |
| $R_{AM}$ SPAD unit, 69 das | Ionome | 15 | 27 | 0.169 | 0.029 | 0.467 | 0.001 | 0.011 |
| $R_{AM}$ Sr concentration | All | 15 | 32 | -0.026 | -0.089 | 0.433 | 0.146 | 0.249 |
| $R_{AM}$ Sr concentration | Growth | 15 | 6 | -0.044 | -0.150 | 0.767 | 0.184 | 0.289 |
| $R_{AM}$ Sr concentration | Ionome | 15 | 26 | -0.031 | -0.095 | 0.435 | 0.153 | 0.254 |
| $R_{AM}$ V concentration | All | 15 | 32 | 0.055 | -0.076 | 0.498 | 0.018 | 0.064 |
| $R_{AM}$ V concentration | Growth | 15 | 6 | -0.101 | -0.150 | 0.781 | 0.219 | 0.334 |
| $R_{AM}$ V concentration | Ionome | 15 | 26 | 0.041 | -0.086 | 0.455 | 0.019 | 0.065 |
| $R_{AM}$ Zn concentration | All | 15 | 32 | -0.100 | -0.148 | 0.419 | 0.248 | 0.368 |
| $R_{AM}$ Zn concentration | Growth | 15 | 6 | -0.031 | -0.149 | 0.861 | 0.177 | 0.283 |
| $R_{AM}$ Zn concentration | Ionome | 15 | 26 | -0.143 | -0.149 | 0.328 | 0.411 | 0.528 |

**Supplemental Table S6.** Volumetric weight, pH, salt and nutrient content of mixed, unfertilized substrate used in all experiments.

| | substrate (sand:clay; 9:1) | | | |
|---|---|---|---|---|
| volumetric weight (wet) | 1667.7 | ± | 7.8 | g/l |
| pH | 7.6 | ± | 0.1 | |
| salt content | 332 | ± | 3.7 | mg/l |
| conductivity | 37.7 | ± | 0.5 | µS/cm |
| N soluble | 16 | ± | 0 | mg/l |
| N as ammonium | 8 | ± | 0 | mg/l |
| N as nitrate | 8 | ± | 0 | mg/l |
| Mg | 18 | ± | 1.4 | mg/l |
| $P_2O_5$ | 42.7 | ± | 20 | mg/l |
| $K_2O$ | 100.7 | ± | 49 | mg/l |

**Supplemental Table S7.** Data scale selected for statistical analysis. For each trait, models were fitted on the raw and log-transformed scale, and the scale used for statistical analysis was selected based on residual diagnostics and AIC. The table reports the number of observations available for each trait and the selected scale used for statistical analysis.

| trait | n | selected scale |
|---|---|---|
| arbuscules | 87 | log-transformed |
| As concentration | 167 | log-transformed |
| As content | 167 | log-transformed |
| B concentration | 166 | log-transformed |
| B content | 166 | log-transformed |
| Ca concentration | 166 | log-transformed |
| Ca content | 166 | log-transformed |
| Cd concentration | 166 | log-transformed |
| Cd content | 166 | log-transformed |
| Cr concentration | 165 | log-transformed |
| Cr content | 165 | log-transformed |
| Cs concentration | 167 | log-transformed |
| Cs content | 167 | log-transformed |
| Cu concentration | 166 | log-transformed |
| Cu content | 166 | log-transformed |
| Fe concentration | 165 | log-transformed |
| Fe content | 165 | log-transformed |
| gross water use efficiency | 170 | raw |
| growth stage, 58 das | 170 | raw |
| growth stage, 69 das | 169 | raw |
| heading date | 147 | log-transformed |
| K concentration | 167 | log-transformed |
| K content | 167 | log-transformed |
| Li concentration | 165 | log-transformed |
| Li content | 165 | log-transformed |
| Mg concentration | 167 | log-transformed |
| Mg content | 167 | log-transformed |
| Mn concentration | 167 | log-transformed |
| Mn content | 167 | log-transformed |
| Mo concentration | 167 | log-transformed |
| Mo content | 167 | log-transformed |
| N concentration | 170 | log-transformed |
| N content | 170 | log-transformed |
| Na concentration | 166 | log-transformed |
| Na content | 166 | log-transformed |
| Ni concentration | 166 | log-transformed |
| Ni content | 166 | log-transformed |
| $NO_3$ concentration | 168 | log-transformed |
| $NO_3$ content | 168 | log-transformed |
| $NO_3$ in total N | 160 | log-transformed |
| P concentration | 167 | log-transformed |
| P concentration | 167 | log-transformed |
| P content | 166 | log-transformed |
| P content | 167 | log-transformed |
| Pb concentration | 166 | log-transformed |
| Pb content | 166 | log-transformed |
| plant height | 170 | raw |
| $PO_4$ concentration | 169 | log-transformed |
| $PO_4$ content | 169 | log-transformed |
| $PO_4$ in total P | 166 | log-transformed |
| Pt concentration | 166 | log-transformed |
| Pt content | 166 | log-transformed |
| Rb concentration | 167 | log-transformed |
| Rb content | 167 | log-transformed |
| root DW | 170 | log-transformed |
| root FW | 170 | log-transformed |
| root water content | 170 | raw |
| S concentration | 170 | log-transformed |
| S concentration | 170 | log-transformed |
| S content | 169 | log-transformed |
| S content | 167 | log-transformed |
| shoot area | 170 | log-transformed |
| shoot DW | 170 | log-transformed |
| shoot FW | 170 | log-transformed |
| shoot water content | 170 | log-transformed |
| shoot:root ratio | 170 | log-transformed |
| $SO_4$ concentration | 170 | log-transformed |
| $SO_4$ content | 169 | log-transformed |
| $SO_4$ in total S | 169 | log-transformed |
| SPAD unit, 58 das | 170 | log-transformed |
| SPAD unit, 69 das | 169 | log-transformed |
| Sr concentration | 165 | log-transformed |
| Sr content | 165 | log-transformed |
| total colonisation | 87 | log-transformed |
| total irrigation | 170 | log-transformed |
| V concentration | 166 | log-transformed |
| V content | 166 | log-transformed |
| vesicles | 87 | log-transformed |
| Zn concentration | 166 | log-transformed |
| Zn content | 166 | log-transformed |

## Supplementary Materials and Methods 1

### Growth conditions

For experiment 1, plants were grown in round 7,5 l pots (21 cm deep, 26 cm wide), lined with microperforated foil and filled with 14 kg of substrate (Table S6), a 9:1 mix of sand (Casafino Quarzsand, fire-dried, particle size 0,7-1,2 mm, BayWa AG, Munich, Germany) and construction-purpose clay (CLAYTEC Baulehm, dried, ground, particle size <0,5mm, CLAYTEC GmbH & Co. KG, Viersen, Germany). Plants received a starting dose of 1,3 g of Hauert Plantaktiv 18-0-22 NK fertilizer (Hauert HBG Dünger AG, Grossaffoltern, Switzerland). Prior to sowing, seeds were sterilized with 4 % NaOCl for 3 minutes, thoroughly rinsed and imbibed overnight in sterile water. Each pot was watered with 900 ml of water to roughly 60 % water holding capacity.

Four seeds per pot were sown in 2 cm depth. AM-treated pots were inoculated in the planting hole at 6 cm depth with 1000 Rhizophagus irregularis spores per seed (4000 per pot), followed by watering with 100 ml of water. The spore inoculum was prepared according to Torabi et al. (2021).

16 days after sowing (das) plants were reduced to one plant per pot. The greenhouse temperature controls were programmed to ventilate beyond a maximum of 22°C and heat below a minimum of 16°C. Daily supplemental lighting (48 sodium-vapor lamps, Philips Son-T AGRO; 400 W each) produced 30 kLux. Starting with 7h per day, lighting was gradually increased to 8, 9, 10, 11, 12, 13, 14, 15 hours per day at 30, 33, 34, 36, 37, 40, 41, 42 das, respectively. Plants were watered daily to 60 % water holding capacity, and to 72 % starting from 30 das. Images were collected on a daily basis between thinning and harvest. During the experiment, plants were fertilized twice (29 & 43 das) with 100 ml of 1,4 % Hauert Plantaktiv 18-0-22 NK and 0,1 % Wuxal® Multimicro fertilizer (Hauert MANNA Düngerwerke GmbH, Nürnberg, Germany). Although the main fertilizer is labelled as phosphorus-free (18-0-22), laboratory analysis revealed residual phosphorus content. Based on these measurements, a total of 25 mg phosphorus per plant was applied via fertilization. In addition, the substrate contained approximately 156 mg phosphorus per pot, based on a chemical analysis of the substrate. This resulted in a maximum total phosphorus availability of approximately 181 mg per plant over the course of the experiment.

For experiment 2, maize seeds were sterilized as for experiment 1 before being placed on wet germination paper in a square petri dish. Seeds were kept in the dark for two days in a growth chamber at room temperature. After two days, seedlings were introduced to a 16 / 8 h day/ night cycle. Seedlings were transplanted into 5,5 l pots containing 7 kg of the 9:1 sand:clay substrate used for experiment 1 at five das. A round disc of microperforated foil identical to the one use in experiment 1 was placed at the bottom of the pot before filling and 1,2 g of Hauert

Plantaktiv 18-0-22 NK fertilizer per pot was mixed into the substrate. Before the transfer of the seedlings, each pot received 400 ml of water. During transfer, seedlings were inoculated with 2000 R. irregularis spores per plant 4 cm below the seedling. Directly after transfer each pot was watered with an additional 100 ml of water.

Pots were transferred to a greenhouse with a Lemnatec system with 16 h supplemental lighting producing 350 $\mu mol \cdot m^{-2} \cdot s^{-1}$ per day. Day/ night temperature was 21 / 17 °C and was increased to 26 / 19 °C at 19 das. Plants were watered daily to 50 % water holding capacity. Watering was increased to 60 % at 34 das. Additionally, visible light and fluorescence top-/ side-view images of plants were acquired on a daily basis using a LemnaTec-Scanalyzer3D platform (LemnaTec GmbH, Aachen, Germany; Junker et al. 2014). During the experiment, plants were fertilized two times (26 & 47 das) with 200 ml 0,75 % Hauert Plantaktiv 18-0-22 NK fertilizer and 0,05 % Wuxal® Multimicro fertilizer.

Although the main fertilizer is labelled as phosphorus-free (18-0-22), laboratory analysis revealed residual phosphorus content. Based on these measurements, a total of 25 mg phosphorus per plant was applied via fertilization. In addition, the substrate contained approximately 78 mg phosphorus per pot, based on a chemical analysis of the substrate. This resulted in a maximum total phosphorus availability of approximately 103 mg per plant over the course of the experiment.

Experiment 3 was performed between June and August in a greenhouse without supplemental illumination. Temperature was kept above 19 °C with heating. Above 21 °C the ceiling was automatically partially opened to ventilate the greenhouse. Additionally, shadowing was used above 20 kLux of external light to reduce heat accumulation.

Maize seeds were prepared as described for experiment 1. Prior to sowing, all pots were watered with 1000 ml of water to roughly 55 % water holding capacity of the substrate. 4 seeds per pot were sown in round 12 l pots 22,9 cm deep and 31 cm wide (TEKU Container 5° MCD 31; Pöppelmann GmbH & Co. KG Kunststoff-Werkzeugbau, Lohne, Germany), lined with a fleece (Climatex 30, Rupert Schaubeck e.K., Aiglsbach, Germany), containing 15 kg substrate. The substrate was identical to the substrate used in experiment 1 and 2. During sowing, AM-treated plants were inoculated with 300 spores per plant as described for experiment 1. Plants were singled to one plant per pot at 8 das. Plants were fertilized three times over the course of the experiment with 200ml 0,9 % Hauert Plantaktiv 18-0-22 NK fertilizer and 0,018 % Wuxal Multimicro fertilizer each.

Although the main fertilizer is labelled as phosphorus-free (18-0-22), laboratory analysis revealed residual phosphorus content. Based on these measurements, a total of 33 mg phosphorus per plant was applied via fertilization. In addition, the substrate contained

approximately 167 mg phosphorus per pot, based on a chemical analysis of the substrate. This resulted in a maximum total phosphorus availability of approximately 200 mg per plant over the course of the experiment. Throughout the experiment the pots were watered every other day to 70 % water holding capacity.

## Supplementary Materials and Methods 2

### Statistical analyses and data visualization

Data import and export were managed using the readxl (Wickham and Bryan 2025), openxlsx (Schauberger and Walker 2025) and writexl packages (Ooms 2024). Data manipulation and reshaping were conducted using dplyr (Wickham et al. 2023), tidyr (Wickham et al. 2024), tibble (Müller and Wickham 2023), purrr (Wickham and Henry 2026) and Rmisc (Hope 2022). Figures were generated using ggplot2 (Wickham 2016), with ggh4x used for facet layout adjustments and panel sizing (van den Brand 2024), cowplot for figure assembly (Wilke 2024), ggpubr for annotations (Kassambara 2023), ggrepel for positioning of text labels (Slowikowski 2026), corrplot for correlation matrices (Wei and Simko 2021) and extrafont for font handling (Chang 2023).

Mixed-effects models were fitted using lme4 (Bates et al. 2015) and p-values for fixed effects were obtained using lmerTest (Kuznetsova et al. 2017). Estimated marginal means and contrasts were calculated using emmeans (Lenth and Piaskowski 2025). Pairwise and non-parametric tests were conducted using rstatix (Kassambara 2025). Homogeneity of variance was assessed using Brown–Forsythe tests implemented in the car package (Fox and Weisberg 2019).

For quantitative traits, statistical tests were performed either on the raw or on the log-transformed scale. This choice was made based on residual diagnostics from linear models fitted on both scales. Model residuals were evaluated using Q-Q plots, Shapiro-Wilk tests, Brown-Forsythe tests for variance homogeneity and AIC, and the scale with the better residual behavior was used for inference. Ion concentration and ion uptake traits were analysed on the log scale throughout, whereas growth stage traits were analyzed on the original scale using non-parametric tests (Table S7).

For pairwise comparisons between treatment groups, we performed independent two-sample t-tests, except for growth stage measurements, for which a Mann-Whitney U Test was applied due to the ordinal data. Analysis of Variance (ANOVA) was used for multi-group comparisons, followed by Tukey’s HSD post hoc tests to identify specific differences between groups using the agricolae package (De Mendiburu 2023). Unless otherwise stated, all tests were evaluated at a significance level of $\alpha = 0.05$. P-values were adjusted for multiple testing using the false discovery rate (FDR) where applicable.

Data derived from images taken over the course of the experiment (experiment 1 and 2) were analyzed using linear mixed-effects models. In both experiments, genotype, treatment and day after sowing, as well as all interactions among these factors, were included as fixed effects. Day after sowing was modelled as a categorical factor, allowing treatment contrasts to be

estimated separately at each measured time point. Repeated measurements were accounted for by including plant identity as a random intercept term. Treatment effects at each genotype-by-time combination were estimated from model-derived estimated marginal means. The first significant AM effect was defined as the first measured day at which the AM versus control contrast was significant after FDR correction.

Pearson correlation analyses were used to assess associations between traits. Correlation coefficients, two-sided p-values, sample sizes and 95% confidence intervals were calculated using complete observations. Where applicable, correlations were calculated separately by treatment; correlations involving colonization traits were restricted to inoculated plants.

To assess whether $R_{AM}$ of individual traits could be predicted from $R_{AM}$ of other traits, we performed an exploratory multivariate predictive analysis on genotype means from experiment 1. For each genotype and trait, $R_{AM}$ was calculated as the difference between the AM and control mean. Ridge regression models were fitted using the glmnet package (Friedman et al. 2010) with alpha = 0. Each $R_{AM}$ trait was used once as a response variable and was predicted either from all remaining $R_{AM}$ traits, only from growth-related $R_{AM}$ traits, or only from ionome-related $R_{AM}$ traits. If the response trait was part of the respective predictor set, it was excluded from the predictor matrix. Predictor and response variables were centered and scaled prior to modelling, and internal standardization in glmnet was disabled. Predictive performance was quantified as leave-one-out cross-validated $R^2$. Statistical support was assessed by permutation testing with 1000 random permutations of the response variable, followed by Benjamini-Hochberg correction for multiple testing. Bootstrap resampling with 1000 iterations was used to derive exploratory 95% intervals for $R^2$. Because the analysis was performed on genotype means across 15 lines, it was interpreted as exploratory and used to assess cross-trait predictability rather than to infer causal, mechanistic or genetic relationships.

## References


Bates D, Mächler M, Bolker B, Walker S (2015) Fitting linear mixed-effects models using lme4. J Stat Softw 67 (1). doi:https://doi.org/10.18637/jss.v067.i01

Chang W (2023) extrafont: tools for using fonts.

De Mendiburu F (2023) agricolae: statistical procedures for agricultural research.

Friedman J, Hastie T, Tibshirani R (2010) Regularization paths for generalized linear models via coordinate descent. J Stat Softw 33 (1):1–22. doi:https://doi.org/10.18637/jss.v033.i01

Hope RM (2022) Rmisc: ryan miscellaneous.

Junker A, Muraya MM, Weigelt-Fischer K, Arana-Ceballos F, Klukas C, Melchinger AE, Meyer RC, Riewe D, Altmann T (2014) Optimizing experimental procedures for quantitative evaluation of crop plant performance in high throughput phenotyping systems. Frontiers in Plant Sciences 5 (1664-462X (Print)):770. doi:https://doi.org/10.3389/fpls.2014.00770

Kassambara A (2023) ggpubr: „ggplot2“ based publication ready plots.

Kassambara A (2025) rstatix: pipe-friendly framework for basic statistical tests.

Kuznetsova A, Brockhoff PB, Christensen RHB (2017) lmerTest package: tests in linear mixed effects models. Journal of Statistical Software 82 (13). doi:https://doi.org/10.18637/jss.v082.i13

Lenth R, Piaskowski J (2025) emmeans: estimated marginal means, aka least-squares means.

Müller K, Wickham H (2023) tibble: simple data frames.

Ooms J (2024) writexl: export data frames to excel „xlsx“ format.

Schauberger P, Walker A (2025) openxlsx: read, write and edit xlsx files.

Slowikowski K (2026) ggrepel: automatically position non-overlapping text labels with "ggplot2".

Torabi S, Varshney K, Villaécija-Aguilar JA, Keymer A, Gutjahr C (2021) Controlled assays for phenotyping the effects of strigolactone-like molecules on arbuscular mycorrhiza development. In: Prandi C, Cardinale F (eds) Strigolactones: Methods and Protocols. Springer US, New York, NY, pp 157–177. doi:https://doi.org/10.1007/978-1-0716-1429-7_13

van den Brand T (2024) ggh4x: hacks for „ggplot2“.

Wei T, Simko V (2021) R package „corrplot“: visualization of a correlation matrix.

Wickham H (2016) ggplot2: elegant graphics for data analysis. Springer-Verlag New York,

Wickham H, Bryan J (2025) readxl: read excel files.

Wickham H, François R, Henry L, Müller K, Vaughan D (2023) dplyr: a grammar of data manipulation.

Wickham H, Henry L (2026) purrr: functional programming tools.

Wickham H, Vaughan D, Girlich M (2024) tidyr: tidy messy data.

Wilke C (2024) cowplot: streamlined plot theme and plot annotations for „ggplot2“.
